# The Sox2 transcription factor binds RNA

**DOI:** 10.1038/s41467-020-15571-8

**Published:** 2020-04-14

**Authors:** Zachariah E. Holmes, Desmond J. Hamilton, Taeyoung Hwang, Nicholas V. Parsonnet, John L. Rinn, Deborah S. Wuttke, Robert T. Batey

**Affiliations:** 1 Department of Biochemistry, University of Colorado at Boulder, Campus Box 596, Boulder, CO 80309 USA; 2 BioFrontiers Institute, University of Colorado at Boulder, Campus Box 596, Boulder, CO 80309 USA

**Keywords:** DNA, DNA-binding proteins, RNA-binding proteins, Transcription factors, RNA

## Abstract

Certain transcription factors are proposed to form functional interactions with RNA to facilitate proper regulation of gene expression. Sox2, a transcription factor critical for maintenance of pluripotency and neurogenesis, has been found associated with several lncRNAs, although it is unknown whether these interactions are direct or via other proteins. Here we demonstrate that human Sox2 interacts directly with one of these lncRNAs with high affinity through its HMG DNA-binding domain in vitro. These interactions are primarily with double-stranded RNA in a non-sequence specific fashion, mediated by a similar but not identical interaction surface. We further determined that Sox2 directly binds RNA in mouse embryonic stem cells by UV-cross-linked immunoprecipitation of Sox2 and more than a thousand Sox2-RNA interactions in vivo were identified using fRIP-seq. Together, these data reveal that Sox2 employs a high-affinity/low-specificity paradigm for RNA binding in vitro and in vivo.

## Abstract (web summary)

Some transcription factors have been proposed to functionally interact with RNA to facilitate proper regulation of gene expression. Here the authors demonstrate that human Sox2 interact directly and with high affinity to RNAs through its HMG DNA-binding domain.

## Introduction

A significant number of transcription factor-RNA associations have been reported that are proposed to regulate gene expression in multiple ways, suggesting that these interactions are quite prevalent^1,2^. For example, the *PANDA* lncRNA acts as a molecular decoy through its interaction with nuclear transcription factor Y (NF-YA), thereby titrating the transcription factor from its target genes to decrease their expression^3^. The transcription factor Yin Yang 1 (YY1) binds nascent transcripts, increasing its retention at actively transcribed promoters and the subsequent activation of its target genes^4^. p53 can bind to the 5ʹ-untranslated region of *mdmx* mRNA to regulate translation^5^ and its interaction with RNA may regulate oligomerization and DNA-binding activity^6^. RNA can also serve as a scaffold for transcription factor assembly, as proposed for the *HSR1* RNA-dependent trimerization of the heat shock factor 1 (HSF1) transcription factor to activate heat shock-responsive target genes^7^. While an expanding set of transcription factors are being found to associate with the transcriptome^8–15^, the majority of these studies do not distinguish between direct and indirect association of the transcription factor with RNA and little is known about the biochemical or structural basis for the interactions^16^.

A transcription factor of high interest that is also proposed to be associated with RNA is the transcription factor Sox2. Sox2 regulates pluripotency^17,18^ in embryonic stem cells through expression of pluripotency-associated transcription factors and repression of lineage-specific genes^19,20^. Sox2 is essential for embryonic development and its knockout or dysregulation leads to terminal differentiation and embryonic lethality^20–23^. In combination with several other transcription factors, including Oct4^24,25^, Sox2 reprograms somatic cells back to a pluripotent state. Further, induction of pluripotency can be achieved with just Sox2 and Oct4 alone under specific conditions^26^, highlighting the core role Sox2 plays in the maintenance of pluripotency. Sox2’s role further extends throughout neuronal development^27,28^, as Sox2 maintains neural progenitor cells (NPCs) in an undifferentiated, slowly proliferating state by repressing the function of pro-neuronal differentiation proteins^29^. To fully understand how Sox2 governs these processes, the networks of interactions with partners governing its regulation and function are important to elucidate.

In addition to its well-defined roles in direct transcriptional regulation at the DNA level, Sox2 was found associated with lncRNAs involved in maintaining pluripotency^30,31^ and promoting neuronal differentiation^31,32^. The lncRNAs *ES1* (AK056826) and *ES2* (EF565083) are proposed to regulate pluripotency in a Sox2-dependent manner based on stem-cell specific expression, downregulation upon differentiation, and changes to the global transcriptome upon knockdown that were similar to *Sox2* RNAi^31^. Association between Sox2 and these lncRNAs is based upon a pull-down-type experiment in which Sox2 was immunoprecipitated and the *ES1* and *ES2* transcripts quantified in the bound fraction, although whether this is direct or mediated via other proteins was not determined^31^. This type of analysis also identified a Sox2 association with the rhabdomyosarcoma 2-associated transcript (*RMST*), a lncRNA proposed to regulate the Sox2-dependent expression of neuronal differentiation genes^32^. More recently, analyses of the protein interactome of Sox2 in mouse embryonic stem cells identified a number of RNA-binding protein partners, indicating the potential for broader association of Sox2 with the transcriptome^33^. A separate approach that identifies proteins involved in ribonucleoprotein (RNP) complexes also implicated Sox2 as associating with RNA, but again, whether this association is direct or indirect remains unknown^34^.

To determine the nature of the association of Sox2 with RNA, we investigated its ability to directly interact with RNA both in vitro and in vivo. In this study, we measured the binding affinity of Sox2 for *ES2* lncRNA using fluorescence anisotropy (FA) supplemented with an electrophoretic mobility shift assay (EMSA). We observed that Sox2 binds *ES2* directly with high affinity through its DNA-binding HMG domain. Using a minimization approach and a library of RNA hairpins designed to test structural contributions to affinity, we found that Sox2-HMG does not bind a single specific site within *ES2* but rather generally binds RNAs with a high degree of double-stranded character. To further define the RNA-binding characteristics of the Sox2-HMG domain, we compared the binding modes of DNA and RNA using biochemical and biophysical approaches including NMR spectroscopy and alanine scanning mutagenesis. These data revealed that the HMG domain uses the same surface to bind DNA and RNA, but only a partially overlapping set of amino acids are used to bind each with high affinity. To complement the in vitro biochemical data, we used formaldehyde RNA immunoprecipitation (fRIP) and UV-RNA immunoprecipitation (UV-RIP) in mouse embryonic stem cells (mESC) to establish that Sox2 directly interacts with RNA in vivo. Collectively our data determine that Sox2 directly binds RNA in vitro and in vivo—with high-affinity and low-specificity substrate properties.

## Results

### Sox2 directly binds *ES2* RNA in vitro with high affinity

**Fig. 1:**
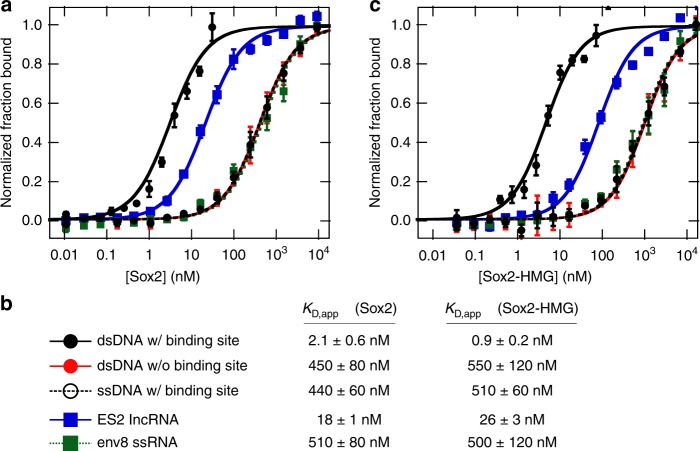
Nucleic acid binding by Sox2 and Sox2-HMG. **a** Average binding curves of full-length Sox2 to nucleic acid ligands. Curves are presented as the average of normalized fraction bound with error bars displaying the standard deviation. **b** Figure legend and measured binding affinity values for panels (**a**) and (**c**). Values are presented as the average and s.e.m. of *n* = 4 technical replicates with the exception of Sox2-HMG/*ES2* which is the average and s.e.m. of *n* = 10 technical replicates. **c** Representative binding curves of the Sox2-HMG domain to nucleic acid ligands. Curves and error bars displayed as in panel (**a**).

While Sox2 has been observed indirectly associated with several lncRNAs^31,32,35^, a direct interaction has yet to be established. To unambiguously determine whether Sox2 directly binds RNA, we used fluorescence anisotropy (FA) to monitor binding in vitro with highly purified components. The interaction of Sox2 with a fluorescein-labeled DNA containing the *FGF4* enhancer element (sequences of all DNAs used in this study are shown in Supplementary Table 1 and RNA sequences in Supplementary Table 2), which contains a well-characterized Sox2-binding sequence^36^, yielded an apparent equilibrium dissociation constant (*K*_D,app_) of 2.1 ± 0.6 nM ((*n* = 4) standard error of the mean (s.e.m.)) for the high-affinity transition (Fig. 1a, b), which is well within the range of prior measurements (0.4 nM^37^ to 15 nM^38,39^). As evidence for site-specific DNA binding, Sox2 binds poorly to single-stranded DNA containing the consensus site and DNA lacking the consensus site. Each of these ligands binds with >200-fold lower affinity than the *FGF4* enhancer sequence (Fig. 1a, b).

To assess direct interaction between Sox2 and RNA, binding to the *ES2* lncRNA (354 nucleotides (nts)^31^) was performed. This RNA was selected for initial study due to the experimental tractability of its short length compared with other proposed interacting transcripts^31,32,35^ (e.g., *RMST*, 2.5 kb). The FA assay reveals that Sox2 binds *ES2* with a *K*_D,app_ of 18 ± 1 nM (*n* = 8, s.e.m.) (Fig. 1a, b). This is a modestly weaker affinity than observed for consensus DNA binding but significantly tighter than non-specific binding to ssDNA and non-consensus DNA. Binding was also assessed to a ssRNA consisting of five repeats of the sequence 5ʹ-AUACAAC, which lacks stable secondary structure^40^. Sox2 binds this RNA with an affinity equivalent to that of ssDNA and non-consensus DNA (Fig. 1a, b). These data reveal that Sox2 can directly interact with RNA in vitro and that its RNA-binding activity has structural and/or sequence requirements.

### The Sox2-HMG domain mediates interactions with RNA

As the only known nucleic acid-binding domain within Sox2^41^, we hypothesized that the HMG domain was responsible for interaction with RNA. The binding affinity of the Sox2-HMG domain (amino acids 40–123) was evaluated for the same nucleic acids described above. We observe that Sox2-HMG fully recapitulates the binding profile exhibited by the full-length protein (Fig. 1b, c). The agreement between these two data sets clearly demonstrates that the Sox2-HMG domain is fully responsible for mediating interactions with both DNA and RNA. Further, the magnitude of the measured binding affinity for *ES2* supports its biological relevance, as the *K*_D,app_ falls within the range of observed affinities for experimentally validated Sox2 sites in the genome (Supplementary Table 1)^37,42,43^.

### Sox2-HMG binds several sites in *ES2* RNA with high affinity

**Fig. 2:**
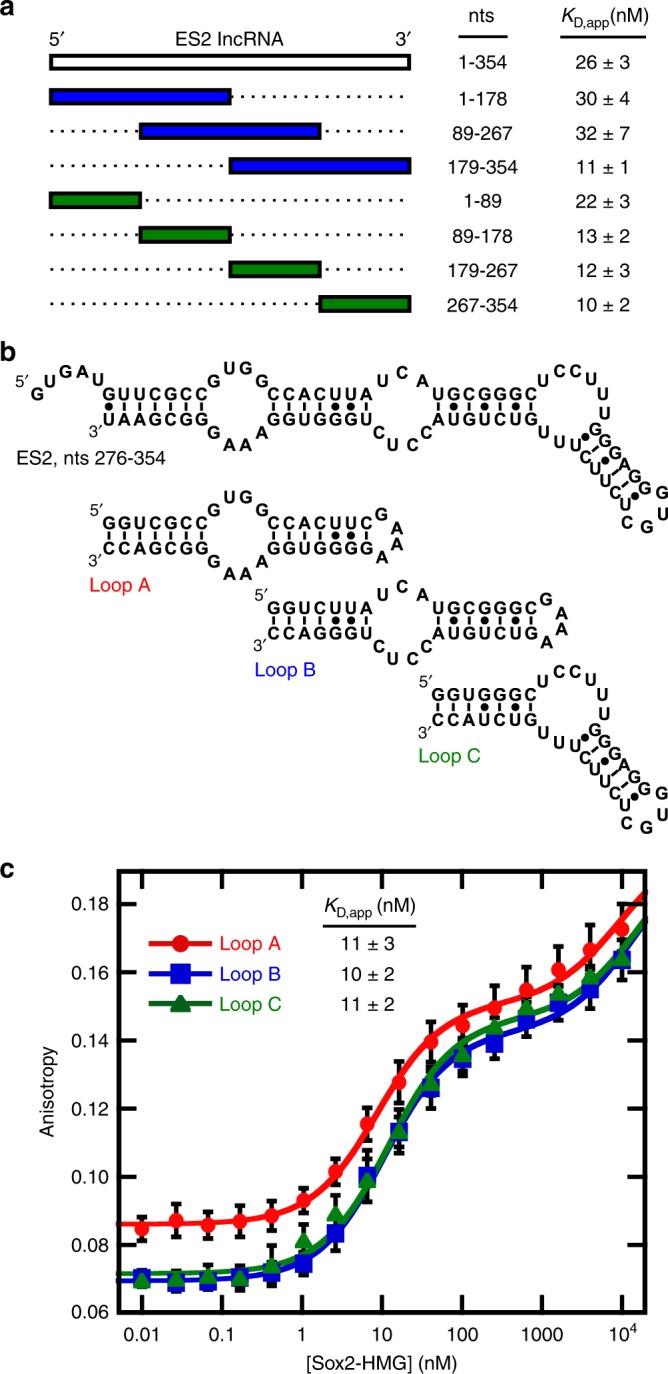
Deletion analysis of the *ES2* lncRNA. **a** Schematized depiction of the *ES2* lncRNA and the segmented transcripts. Apparent binding affinity displayed as the average and s.e.m. of *n* = 6 technical replicates excluding 1–354 ES2, which is *n* = 10 technical replicates). **b** Sfold^44^ predicted secondary structure of *ES2*, nts 276–354, and minimized loop constructs. **c** Average binding curve for each Sox2-RNA interaction with error bars displaying the standard deviation of each measurements. *K*_D,app_ is reported for the first transition and presented as the average and the s.e.m. of *n* = 6 technical replicates.

An unbiased minimization approach was taken to identify a potential specific Sox2-binding site within *ES2* lncRNA. The secondary structure of *ES2* in the cellular context is unknown, preventing a structure-based deletion analysis approach. Instead, the full-length RNA was segmented using two distinct strategies into either three overlapping or four nonoverlapping equally sized segments (Fig. 2a) and the apparent binding affinities to this set of RNA constructs was measured. Unexpectedly, we find that the Sox2-HMG domain binds all *ES2* fragments with nearly the same affinity (Fig. 2a). The ability of Sox2 to bind all fragments suggests that it does not have a single, discrete binding site within *ES2*, but rather interacts with a common RNA structural element.

To further define the RNA sequence and/or structure features that the Sox2-HMG domain binds, a representative fragment of *ES2* (nts 267–354) was further divided into three RNAs that preserve both the sequence and computationally predicted secondary structure of the parental RNA element (Loops A–C; Fig. 2b)^44^. These fragments constitute a set of overlapping RNA hairpins, each centered upon a single internal loop flanked by the paired regions and capped with either a GAAA or GUGC tetraloop. Sox2-HMG binds each of these *ES2*-derived hairpins with equivalent affinity (Fig. 2c), indicating that Sox2 does not recognize RNA in a sequence-specific fashion.

### Sox2 prefers to interact with double-stranded RNA elements

**Fig. 3:**
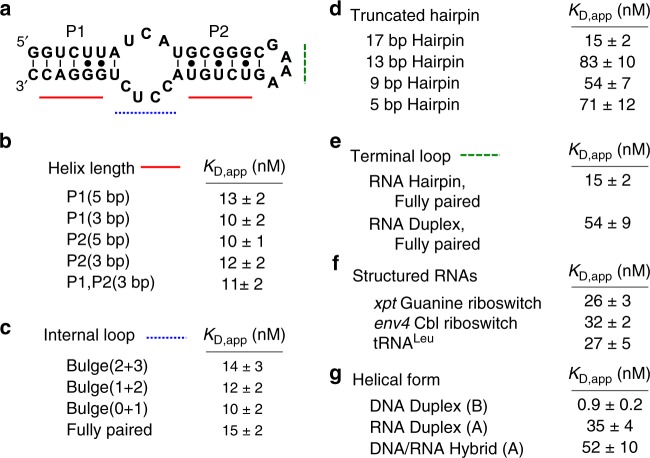
Interrogation of RNA features for high-affinity interaction with Sox2-HMG. **a** Secondary structure of the Loop B RNA with the designated paired regions (P1 and P2), internal loop (blue dotted line), and terminal loop (green dashed line). Binding assessed to mutations **b** reducing paired region helix length, *n* = 8, **c** reducing the internal loop size, *n* = 8, **d** truncation of hairpins, *n* = 3 **e** removing the terminal loop, *n* = 6, **f** structured RNAs, xpt riboswitch and env4 Cbl riboswitch, *n* = 4, tRNA^Leu^, *n* = 3, and **g** nucleic acid duplexes with *n* = 3 technical replicates each. Apparent binding affinity displayed as the average with s.e.m. reported.

To further delineate the structural requirements for high-affinity binding by Sox2-HMG, mutations were made to the Loop B RNA identified in the *ES2* deletion analysis (Fig. 3a, secondary structures provided in Supplementary Fig. 3). Reducing the length of either or both paired regions by two base pairs did not substantially impact observed binding affinity (Fig. 3b). To assess the possibility that Sox2-HMG recognizes the internal loop region of the hairpin RNA, a series of mutations were made that systematically convert the internal loop to duplex RNA (Fig. 3c). Again, no detectable difference in Sox2-HMG binding affinities was observed between these bulged and duplex RNAs, suggesting that a hairpin is all that is required for Sox2-HMG/RNA binding. To identify the minimal RNA hairpin that supports Sox2-HMG binding, we used three truncated hairpins derived from the fully paired internal loop with a sequential reduction of four base pairs down to a five base-pair hairpin. While the affinity was reduced compared with some of the other RNAs, binding affinities tighter than 100 nM were still observed (Fig. 3d). These data reveal that Sox2-HMG can interact with hairpins of the size that are likely to be present in most RNA transcripts.

To examine whether Sox2 exclusively binds hairpin motifs, we interrogated the terminal loop that caps each of the hairpins. Binding between Loops A, B, and C are practically indistinguishable (Fig. 2c) although Loop A and B RNAs have a GAAA tetraloop whereas Loop C has a GUGC terminal loop. To assess contributions of the tetraloop/terminal loop, the Loop B and Loop B/fully paired RNAs were recreated as a two-piece RNA duplex. The two-piece Loop B RNA was unable to anneal with high efficiency, precluding characterization, but Loop B/fully paired readily formed the appropriate duplex structure. Sox2-HMG exhibited a 3.5-fold decrease in binding affinity for the Loop B/fully paired duplex lacking the tetraloop (Fig. 3e). Together, these data indicate that Sox2-HMG principally interacts with A-form double-stranded regions of RNA with other features, such as internal and terminal loops, playing minor or negligible roles in promoting protein–RNA interactions.

Based upon the above findings, we hypothesized that Sox2-HMG binds other RNAs and A-form helices with similar affinities. Toward this end, we first measured the apparent binding affinity of Sox2-HMG for three highly structured RNAs derived from bacteria: two riboswitches and tRNA^Leu^ (Fig. 3f). Remarkably, these RNAs bind Sox2-HMG with affinities comparable to the *ES2* lncRNA. To further test the hypothesis that Sox2-HMG primarily recognizes A-form helices, we prepared a hybrid DNA/RNA duplex based upon the Loop B/fully paired RNA duplex in which the unlabeled RNA strand was replaced by the equivalent DNA. Sox2-HMG binds this hybrid duplex with a comparable affinity (52 ± 10 nM (*n* = 3, s.e.m.)) as the RNA duplex (Fig. 3g), providing additional evidence that Sox2-HMG broadly recognizes A-form nucleic acids. Together, these data indicate that Sox2-HMG should be considered, in addition to being a transcription factor, an RNA-binding protein. In addition to interacting with DNA, this activity would enable Sox2 to also interact with a broad spectrum of RNAs in the cell.

### DNA and RNA binding to Sox2-HMG is mutually exclusive

**Fig. 4:**
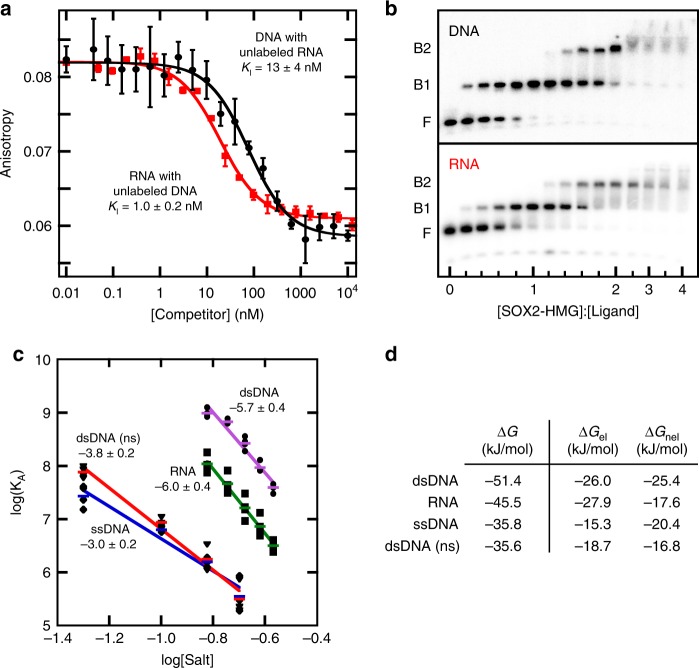
Characterizing the nucleic acid-binding modes of Sox2-HMG. **a** Equilibrium competition binding assays measuring the anisotropy of the labeled nucleic acid as a function of unlabeled competitor. Binding curves presented as the average of *n* = 3 technical replicates with the error bars reflecting their s.d. **b** Determination of complex stoichiometry by EMSA. Migration of the radiolabeled nucleic acid species as a function of [Sox2-HMG]:[Ligand]. Bands are denoted as F (free), B1 (bound species 1), and B2 (bound species 2). Quantification provided in Supplementary Fig. 5. **c** Analysis of the salt dependence of the Sox2-HMG/nucleic acid interactions. The colored bars show the measured binding affinities as the average of the *n* = 4 with the standard error reported (black shapes). The calculated slope of each linear regression is provided. **d** The calculated values of the electrostatic (Δ*G*_el_) and non-electrostatic (Δ*G*_nel_) components to binding the indicated nucleic acid ligand under the standard reaction conditions.

To determine if DNA and RNA binding to Sox2 are mutually exclusive, equilibrium competition binding experiments were performed by titrating fluorescently labeled nucleic acid/protein complex with increasing amounts of unlabeled nucleic acid competitor. For these experiments we used two high-affinity nucleic acid ligands: the DNA duplex containing the *FGF4* enhancer Sox2-binding site and Loop B/Bulge(0 + 1) hairpin RNA. DNA and RNA competitively bind Sox2-HMG with apparent inhibition constants (*K*_I,app_) consistent with directly measured dissociation constants (DNA_FAM_ with RNA_unlabeled_ = 13 ± 4 nM (*n* = 3, s.e.m.); RNA_FAM_ with DNA_unlabeled_ = 1.0 ± 0.2 nM (*n* = 3, s.e.m.)) (Fig. 4a). As a control, the observed anisotropy of the fluorescently labeled ligand does not change while titrating unlabeled nucleic acid in the absence of Sox2-HMG (Supplementary Fig. 4). Moreover, self-competition between fluorescently labeled and unlabeled nucleic acid ligands yield apparent inhibition constants consistent with binding affinities as determined by direct titrations (Supplementary Fig. 4). Together, these data indicate that Sox2-HMG uses the same or largely overlapping surfaces to interact with DNA and RNA.

### Sox2-HMG forms 1:1 and 2:1 complexes with both DNA and RNA

While the Sox2-HMG domain engages site-specific DNA as a monomer, it is possible that RNA binding is achieved as dimer or other higher-order multimer. The binding stoichiometry of Sox2-HMG to DNA and RNA was assessed using an electrophoretic mobility shift assay where the concentration of labeled nucleic acid greatly exceed the *K*_D_ (Fig. 4b). In this assay, Sox2-HMG shows similar behavior in binding DNA and RNA with two discrete binding transitions. The first bound complex (B1, Fig. 4b) for each nucleic acid saturates at a 1:1 protein:RNA ratio while the second bound state (B2, Fig. 4b) corresponds to a 2:1 protein:RNA complex (Supplementary Fig. 5). The B1 complex corresponds to the high-affinity interaction observed for either ligand and is consistent with the 1:1 stoichiometry observed for the Sox2-HMG:DNA in solution and crystal structures^45,46^. The second shift represents a weak non-specific binding event such as that observed for non-consensus DNA ligands and present at high protein concentrations in equilibrium binding measurements for high-affinity DNA and RNA ligands (>1 µM Sox2-HMG domain). Thus, the stoichiometric binding properties of Sox2 to DNA and RNA are the same.

### Sox2 recognizes RNA and DNA with similar electrostatics

Structures of Sox2-HMG/DNA complexes revealed extensive use of electrostatic interactions to facilitate binding^45,46^; if the Sox2-HMG domain recognizes RNA in a similar fashion as site-specific DNA, then it is expected to be similarly electrostatically driven. To assess the electrostatic contributions to nucleic acid binding, the salt dependence of Sox2-HMG/nucleic acid interactions was determined by measuring *K*_D,app_ as a function of monovalent salt concentration (Fig. 4c)^47^. For the *FGF4* enhancer sequence, we observed a log-linear behavior over a range of NaCl concentrations from 0.15 to 0.27 M. The magnitude of the slope associated with binding is −5.7 ± 0.4 (*n* = 4, standard error), corresponding to 8.2 counterions released. This observation is consistent with the 9 basic amino-acid side chains within 5 Å of the phosphate backbone in a crystal structure^45^ and 10 of these type of contacts present in an NMR structure of the Sox2-HMG/DNA complex^46^. Using a similar approach for RNA binding, we observed a slope of −6.0 ± 0.4 (*n* = 4, standard error), corresponding to 8.7 released counterions. This result indicates that Sox2-HMG uses a similar number of electrostatic interactions to contact DNA and RNA. By comparison, the slopes associated with binding ssDNA and non-specific DNA are substantially less (−3.0 ± 0.2 (*n* = 4, standard error) and −3.8 ± 0.2 (*n* = 4, standard error), respectively), suggesting that the mode of RNA recognition is more similar to that of site-specific DNA binding than to that of non-specific binding. Separating electrostatic (Δ*G*_el_) and non-electrostatic (Δ*G*_nel_) components of binding^48^ reinforces this conclusion, as DNA and RNA have a similar Δ*G*_el_ that is significantly larger than ssDNA and non-specific DNA (Fig. 4d). However, Δ*G*_nel_ for RNA binding more closely aligns with ssDNA and non-specific DNA. Further, the reduced Δ*G*_nel_ contribution to RNA binding supports the observation that the Sox2-HMG domain binds RNA with no apparent sequence specificity.

### Sox2-HMG is partially ordered upon RNA complex formation

**Fig. 5:**
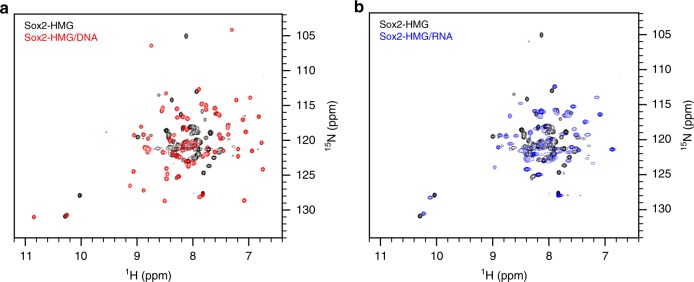
Structure analysis of Sox2-HMG/nucleic acid-binding modes. **a** ^1^H–^15^N HSQC NMR spectrum of Sox2-HMG (black) overlaid with the spectrum of the Sox2-HMG/DNA complex (red). The DNA sequence used in this experiment was derived from the *FGF4* enhancer, corresponding to the sequence shown in Supplementary Table 1. **b** ^1^H–^15^N HSQC NMR spectrum of Sox2-HMG (black) overlaid with the spectrum of the Sox2-HMG/RNA complex (blue). The RNA sequence used in this experiment was the hairpin Loop B/Bulge(0 + 1) (Fig. 3c).

To evaluate the structural features of the Sox2-HMG/RNA complex, we collected and compared ^1^H–^15^N HSQC NMR spectra of ^15^N-labeled Sox2-HMG in the free, DNA- and RNA-bound states. The nucleic acid ligands used for these experiments were a DNA duplex corresponding to the *FGF4* enhancer, Loop B/Bulge(0 + 1), and a fully base-paired RNA hairpin (Supplementary Fig. 3). In the absence of ligand, the Sox2-HMG domain appears loosely structured, as there are discrete crosspeaks on the periphery of a set of resonances characterized by poor dispersion. Upon binding a specifically recognized DNA ligand, the resonances become uniformly narrow and well-dispersed, indicative of the formation of a discrete ordered structure (Fig. 5a), consistent with disorder-to-order transitions observed for other sequence-specific HMG domains^49–51^. In contrast, the Sox2-HMG/RNA complex exhibits spectral properties that is in between the unbound and DNA-bound protein (Fig. 5b). While there remain a number of broad and poorly dispersed resonances as observed in the free protein, a subset of crosspeaks become significantly sharper and well resolved, suggesting that the Sox2-HMG domain becomes partially ordered upon RNA binding. This notable difference from DNA complex formation indicates that the Sox2-HMG engages DNA and RNA differently.

### Distinct residues of Sox2-HMG interact with DNA and/or RNA

**Fig. 6:**
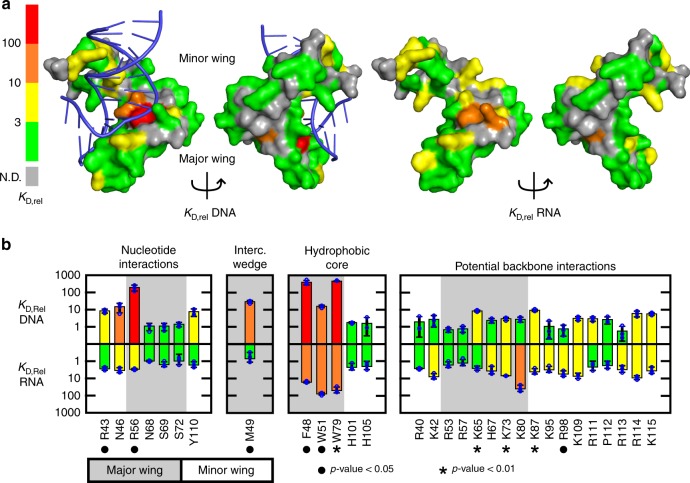
Characterization of the base-specific contributions to nucleic acid binding. **a** The relative binding affinities (*K*_D,rel_) for DNA and RNA are mapped to the Sox2-HMG surface (PDB ID 1GT0) according to the color scale to the left. **b** Plotted *K*_D,rel_ values for DNA and RNA binding colored according to the scale in panel (**a**). The values of *K*_D,rel_ are presented as the average and s.d. of *n* = 3 technical replicates. Bars above a gray background correspond to the Sox2-HMG major wing whereas a white background denotes the Sox2-HMG minor wing. *P*-values for the difference between *K*_D,rel_(DNA) and *K*_D,rel_(DNA) were calculated by the Student’s *t*-test assuming non-parametric distribution. All measured binding affinities and *p*-values for the alanine mutagenesis are provided in Supplementary Table 7.

A comprehensive alanine scan of the Sox2-HMG domain was performed to define and compare the amino acids that make thermodynamic contributions to DNA and RNA recognition. Amino acids in Sox2-HMG known to interact with specific site DNA^45,46^ or that have a known propensity to interact with nucleic acids were selected for analysis^52^. This led to the selection of 48 out of 84 residues in the Sox2-HMG domain for mutation to alanine, a set which covered nearly the complete surface of the protein. The affinity of each mutant in the Sox2-HMG domain for DNA and Loop B/Bulge(0 + 1) hairpin RNA was measured using the FA assay; a range of *K*_D,app_ values from 0.7 ± 0.2 (*n* = 3, standard deviation (s.d.)) to 590 ± 30 (*n* = 3, s.d.) nM were observed. To use this data to visualize the DNA and RNA-binding surface of the HMG domain (an annotated cartoon of the structure of the Sox2-HMG/DNA complex is provided in Supplementary Fig. 6 for reference), the *K*_rel_ (defined as *K*_D,app_(mutant)/*K*_D,app_(wild type)) of each mutant (Supplementary Table 7) was mapped onto the crystal structure of the Sox2-HMG/DNA complex^45^ (Fig. 6a). It should be noted that this visualization onto the DNA-bound structure assumes the Sox2-HMG domain is structured the same way in DNA and RNA complexes, and while this is likely not rigorously accurate in light of the above NMR data, it serves as a useful framework for a comparative analysis.

We find that, while residues important for DNA and RNA-binding cluster on the same face of the protein (Fig. 6, Supplementary Fig. 8), they do not play equivalent thermodynamic roles in DNA and RNA binding. For example, amino acids mediating base-specific DNA contacts play a much greater role in DNA-relative to RNA binding. Specifically, amino acids recognizing the core 5ʹ-dTTGT consensus motif have the greatest contribution to DNA affinity (Fig. 6b). Asn46, which hydrogen bonds with bases at the 5ʹ-TG step, and Arg43 and Tyr110, which interact with the subsequent 5ʹ-GT step, reduce Sox2-HMG DNA-binding affinity approximately 10-fold when substituted with alanine. Additionally, DNA-binding affinity is reduced by substitution of Arg56, which is also believed to be a base-specific interaction^45^. In contrast to DNA binding, RNA binding is only modestly affected by alanine substitution of Asn46 or Arg56 and unaffected by mutation of Arg43 and Tyr110. A second striking difference between DNA and RNA binding is observed for the intercalating Met49 residue that typifies HMG domain binding to the minor groove leading to the characteristic kinking of DNA^53^. Mutation of this residue to alanine substantially reduces DNA binding while maintaining RNA-binding activity. This suggests the characteristic bending function facilitated by intercalation of Met49 is not an essential component of the RNA interaction. Overall, none of the amino acids involved in base-specific contacts to DNA are critical for RNA binding, consistent with the non-sequence specific nature of RNA binding exhibited by the Sox2-HMG.

While we observed that the net number of electrostatic interactions in specific site DNA and RNA binding are the same, mutational analysis reveals that the residues in Sox2-HMG likely mediating these interactions are more important for RNA binding. Moreover, comparison of the contribution of individual surface arginine and lysine residues to binding affinity revealed that the identity of these residues is not completely conserved between the DNA and RNA complexes (Fig. 6). For example, RNA-binding activity is much weaker than DNA binding when Lys80, Lys95, Arg98, or Arg113 is mutated to alanine. In contrast, several basic amino acids, such as Lys87, Lys109, Arg113, and Arg114, have similar effects on both DNA and RNA binding when mutated to alanine. Of the 17 basic residues within the Sox2-HMG domain whose mutation to alanine decreases binding affinity for either DNA or RNA substrate by at least 2.5-fold, 11 of those mutants have a greater impact on RNA binding (Supplementary Table 7), indicating that electrostatic interactions play a larger thermodynamic role in RNA recognition.

In contrast to the differential use of surface residues for DNA and RNA binding, amino acids in the core of the HMG domain have similar impacts upon binding both substrates. Amino acids within the major wing (Phe48, Trp51, and Trp79) have a large impact on binding of DNA and RNA, while several residues involved in the hydrophobic core of the minor wing (His101 and His105) have essentially no effect. These data indicate that the folding and stability of the major wing of the HMG domain is critically important for high-affinity recognition of both DNA and RNA.

### Sox2 interacts with RNA in mESC

**Fig. 7:**
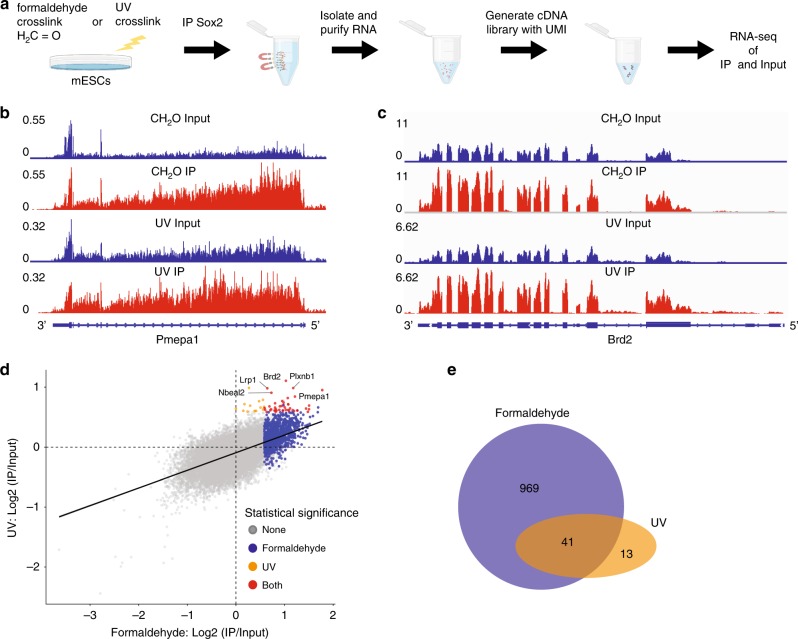
Sox2 interacts with RNA in mESC. **a** Schematic of fRIP and UV-RIP-Seq experimental flow. **b**, **c** Examples of enriched genes (Pmepa1 and Brd2) identified by both formaldehyde (“CH_2_O”) and UV-based RIP-seq. Each panel describes normalized coverage of sequencing reads (RPM: reads per million reads). **d** Correlation of enrichment between fRIP and UV-RIP. A dot represents a gene and each axis describes the fold change of IP relative to input in log scale. A color indicates statistical significance of enrichment (FDR-adjusted *p*-value<0.05): blue significant only by formaldehyde, orange: significant only by UV, red: significant by both formaldehyde and UV. **e** Overlap of genes enriched in both fRIP and UV-RIP. Data for both fRIP (*n* = 4) and UV-RIP (*n* = 4) are obtained from eight total distinct cell culture plates.

Our in vitro analysis demonstrated that Sox2-HMG is a robust RNA-binding domain, raising the question of whether RNA binding also occurs in vivo. To determine if Sox2 has direct RNA interactions, we used ultraviolet (UV) crosslinking immunoprecipitation followed by deep sequencing (UV-RIP-seq), a technique that retrieves directly cross-linked RNA–protein interactions (Fig. 7a)^54^. First, we validated the specificity of the Sox2 antibody and ability to specifically and quantitatively immunoprecipate Sox2 with magnetic beads using western blot (Supplementary Fig. 9). We next performed UV-RIP in which mESC were exposed to UV (254 nm) and RNA that was coprecipitated with the Sox2 antibody was subjected to RNA sequencing analysis. To ensure sequencing reads were not amplification artifacts we employed unique molecular IDs (UMI)^55,56^.

A total of four biological replicates for Sox2 UV-RIP were performed under conditions that maintained pluripotency in mESCs. Each sample was subjected to RNA sequencing to an average read depth of ~20 million reads per sample. To determine if the Sox2 UV-RIP is enriched for specific RNA targets relative to the input, we mapped the sequenced reads using a splicing-aware aligner to the mouse genome (mm38) with gene annotation derived from GENCODE (vM16). A regression-based statistical test was used to identify enriched RNA species in the IP relative to the input. An RNA species was considered to be enriched if it had a >1.5-fold change (FC) and false discovery rate (FDR)-adjusted *p*-value of <0.05. Using this approach, we identified 54 RNA species that were enriched with UV crosslinking, suggestive of Sox2-direct RNA interactions in mESC (Fig. 7b–e).

To confirm the results of UV-RIP as well as potentially observe a more expansive Sox2-RNA interactome, we performed formaldehyde cross-linked immunoprecipitation (fRIP) (Fig. 7a)^57^. fRIP has the advantage of not only detecting direct Sox2-RNA interactions, but also interactions that arise due to proximity to Sox2. Four independent biological replicates of fRIP were performed in which mESCs were cross-linked with 0.1% CH_2_O and subsequently treated using the same methods as in UV-RIP. In contrast to UV-RIP, in fRIP we found many more enriched RNA interactions with Sox2—over a thousand compared with 54 in UV-RIP. Importantly, ~75% of UV-RIP interactions were also detected in fRIP (Fisher exact test: *p*-value < 10^−15^), demonstrating the reproducibility of direct interactions in fRIP as well as a more expansive interactome (Fig. 7e). Consistent with the in vitro analysis, we find that in vivo Sox2 is tightly associated with RNAs.

## Discussion

In this study we demonstrate that Sox2 directly interacts with RNA using its HMG domain. In vitro, the Sox2-HMG domain binds the *ES2* lncRNA and a broad set of smaller RNA hairpins with affinities comparable to that of experimentally validated Sox2 DNA-binding sites. Strikingly, we observed that the Sox2-HMG domain has a tighter affinity for both RNA and RNA–DNA duplexes than non-specific DNA, suggesting a strong non-sequence selective binding mode for nucleic acids with non-B-form helical geometry. Analysis of the NMR structure of the closely related SRY–DNA complex^58^ revealed that protein binding distorts the B-form *MIS* DNA to a helical geometry much more A-form in character than B-form (r.m.s.d. = 2.4 and 4.2 Å, respectively)^59^. This preference for bent DNA is consistent with its role as a pioneer transcription factor, as Sox2 binds compact, inaccessible DNA wrapped in nucleosome structures by recognizing partial DNA-binding sites on the nucleosome surface^37^. The pre-bent structure of nucleosomal DNA is believed to facilitate binding of Sox2 to relaxed consensus sequences through shape complementarity of the widened minor groove^37^. A similar phenomenon is observed for TATA-binding protein (TBP), another minor-groove binding protein that binds pre-bent DNA targets with an affinity 100-fold tighter than the unbent state^60^. The preference of the Sox2-HMG domain for DNA deformed from B-form geometry likely also enables it to bind RNAs with A-form double-stranded elements in a non-sequence-specific fashion with high affinity.

The ability of Sox2 to bind pre-bent nucleosomal DNA has been ascribed to its structural flexibility^37^. We propose that it is this feature that enables Sox2 to interact with high affinity to a spectrum of double-stranded non-B-form nucleic acids. This hypothesis is consistent with the observation that Sox2-HMG, like other HMG domains that bind DNA in a sequence-specific fashion, exists in a weakly structured unbound state^61^. The flexible character of the Sox2-HMG domain would allow it to accommodate a range of nucleic acid geometries. This feature is essential for its duality as a pioneer transcription factor and an RNA-binding protein. Given the proclivity of many HMG domains to also bind non-B-form DNA, it would be anticipated that other HMG domains also have significant RNA-binding activities.

Comparison of the results of the UV-RIP and fRIP experiements reveals a number of associations with the transcriptome via a combination of direct protein–RNA and potentially indirect interactions. Our in vitro and UV-RIP experiments support that Sox2 interacts directly with RNA in vivo. The relatively small set of RNAs captured by this experiment may reflect a limited set of direct Sox2 interaction partners in vivo or the relative inefficiency of crosslinking to double-stranded nucleic acids^62^. On the other hand, fRIP is capable of not only identifying direct interactions, but also those that occur through ribonucleoprotein complexes^33,57^. Our fRIP assay captured ~20-fold more potential Sox2-RNA targets than UV-RIP, suggestive that Sox2 may be also be a component of RNPs. While our in vivo data demonstrate that Sox2-RNA interactions occur broadly within the mESC transcriptome, our findings do not provide information about the mechanism(s) by which RNA binding impacts Sox2 function or vice versa. However, our observation of the substantial association of Sox2 with RNA is consistent with previous studies, all of which point to functional Sox2-RNA interactions in cells^30,31,63^.

Our in vitro studies demonstrate that the Sox2-HMG domain interacts non-sequence specifically with small RNA hairpins. These elements are expected to be a pervasive structural element in RNA, thus it is surprising that Sox2 interacts with a limited set of transcripts in vivo. This selective binding to RNA by Sox2 in vivo may be conferred through interactions with other proteins. The Sox2 proteo-interactome, which involves at least 100 proteins, contains many RNA-binding proteins involved in RNA splicing and processing^33,34,64–66^. For example, Sox2 promotes alternative splicing in transitional carcinoma cells^67^. Together, these data suggest that Sox2 may play multiple roles in RNA metabolism either through direct interaction with RNA or via protein partners.

The high-affinity RNA-binding activity of Sox2 revealed by this study underscores a newly appreciated complex interplay between transcription factors and the transcriptome. The large number of transcription factors identified in unbiased transcriptome-wide protein association surveys points toward the pervasive nature of these interactions^8–15^, although relatively few of these have yet been characterized extensively in vitro. Of those that have, a greater theme of RNA structural recognition emerges in light of the sequence recognition observed for DNA. This departure from classical models of transcription factors requires a reassessment of how these proteins establish proper patterns of gene expression across the genome.

## Methods

### Expression and purification of proteins

A gene encoding Sox2 was cloned into the pET28a vector as an N-terminal hexahistidine fusion using standard molecular biological approaches^68^ and the resultant vector sequence verified (sequences provided in Supplementary Table 10). This plasmid was transformed into BL21(DE3) *E. coli* cells for expression. A saturated overnight culture was inoculated into LB medium supplemented with 50 µg/mL kanamycin at a 1:100 dilution and grown at 37 °C until the OD_600_ reached 0.6. Protein expression was induced at 1 mM isopropyl β-D-1-thiogalactopyranoside (IPTG) at 37 °C for 4 h. The cells were pelleted at 2400 × *g*, resuspended in Sox2 lysis buffer (50 mM Tris, pH 7.5, 300 mM NaCl, 0.5 mg/mL lysozyme), and lysed by flash freezing in liquid nitrogen and thawing at 37 °C three times prior to incubation for 1 h with 20 U/mL DNase I. The resulting lysate was pelleted at 22,500 × *g* and the supernatant discarded. The pelleted inclusion bodies containing Sox2 were washed three times with inclusion body wash buffer (50 mM Tris, pH 7.5, 300 mM NaCl, 0.5% Triton X-100) prior to resuspension in Sox2 denaturing buffer (100 mM Na_2_HPO_4_, pH 8.0, 6 M guanidine hydrochloride, 300 mM NaCl, 10 mM imidazole). The denatured Sox2 solution was then incubated with Ni-NTA agarose (Qiagen) at room temperature (RT) for 30 min. The Sox2-bound Ni-NTA agarose was washed in denaturing high salt buffer (100 mM Na_2_HPO_4_, pH 8.0, 8 M urea, 1 M NaCl, 40 mM imidazole) and eluted with Sox2 denaturing elution buffer (100 mM Na_2_HPO_4_, pH 8.0, 8 M urea, 300 mM NaCl, 300 mM imidazole). Purified protein was flash frozen in liquid nitrogen and stored at −80 °C until ready for use, at which point the protein aliquot was refolded by rapid dilution into a physiological salt buffer (10 mM Tris-HEPES, pH 7.5, 135 mM KCl, 15 mM NaCl).

The Sox2-HMG domain (amino acids 40–123) was cloned into pET15b as an N-terminal hexahistidine fusion. BL21(DE3) *E. coli* cells expressing Sox2-HMG were grown in LB medium supplemented with 100 µg/mL carbenicillin inoculated with a saturated overnight culture, grown at 37 °C until the OD_600_ reached 0.6. Protein expression was induced overnight with 1 mM IPTG at 25 °C. The cells were pelleted at 2400 × *g*, resuspended in Sox2-HMG lysis buffer (50 mM Na_2_HPO_4_, pH 8.0, 300 mM NaCl, 10 mM imidazole), and lysed via C3 cell homogenizer. The lysate was clarified at 22,500 × *g* and incubated with Ni-NTA agarose at 4 °C for 1 h. The bound resin was washed in high salt wash buffer (50 mM Na_2_HPO_4_, pH 8.0, 1 M NaCl, 40 mM imidazole) and eluted in lysis buffer supplemented with 300 mM imidazole. Sox2-HMG was further purified by cleavage of the hexahistidine affinity tag by overnight incubation with bovine alpha-thrombin (4 U/mg, Haematologic Technologies) and separated using a HiLoad 16/600 Superdex G-75 prep grade column (50 mM Na_2_HPO_4_, pH 8.0, 300 mM NaCl) (GE Life Sciences). Fractions containing Sox2-HMG were pooled and dialyzed in physiological salt buffer supplemented with 50% glycerol and stored at −20 °C until use. For the truncated hairpin assays, Sox2-HMG domain was cloned into pET30b as a N-terminal His_8_-MBP fusion. Using a similar purification protocol as above, the MBP tag was removed using HRV-3C protease. This Sox2-HMG domain was verified to have comparable binding affinities as the Sox2-HMG expressed using the prior approach.

Alanine mutations of Sox2-HMG were made using PCR-mediated site-directed mutagenesis^69^. Protein purification and expression was performed using the same protocol as wild type Sox2-HMG but with the Sox2-HMG lysis buffer supplemented with of 0.1 mg/mL lysozyme and cell lysis achieved by three freeze/thaw cycles. Proteins were purified using HisPur Ni-NTA spin columns (ThermoFisher) using the same buffer conditions listed above. The hexahistidine affinity tag was not removed; Sox2-HMG showed no difference in binding with or without the hexahistidine tag.

Isotopically labeled Sox2-HMG for NMR experiments was grown in standard minimal media supplemented with (^15^NH_4_)_2_SO_4_ and purified as described above. Protein concentrations were calculated using the molar extinction coefficients determined using a molar extinction coefficient of 13,980 M^−1^ cm^−1^ for the Sox2-HMG domain, 37,360 M^−1^ cm^−1^ for full-length Sox2, 8480 M^−1^ cm^−1^ for the W51A and W79A mutants of the Sox2-HMG domain and 12,490 M^−1^ cm^−1^ for the Y110A mutant of Sox2-HMG at 280 nm.

### Preparation and purification of nucleic acid ligands

*ES2* lncRNA (EF565083) was cloned into pUC19 using standard restriction cloning techniques^68^ (RNA sequence provided in Supplementary Table 2). Templates for in vitro T7 RNA polymerase transcription of the full length and fragments of *ES2* were amplified by PCR and templates of the shorter transcripts were generated by annealing two complementary oligonucleotides at a final concentration of 500 nM in 0.5x T.E. buffer with 100 mM NaCl by heating to 95 °C and cooling to room temperature at a rate of −2 °C/min. In vitro transcription reactions were performed as previously described^70^ and purified by denaturing polyacrylamide gel electrophoresis. The ssRNA derived from the *env8* cobalamin riboswitch and the FAM-labeled strand of the RNA duplex were chemically synthesized (Integrated DNA Technologies).

RNAs used for FA were labeled at the 3ʹ terminus as described^71^. Briefly, the 3ʹ terminal ribose vicinal diols were oxidized in freshly prepared periodate oxidation buffer (50 mM NaOAc, pH 5.3, 1 mM NaIO_4_) and conjugated to fluorescein-5-thiosemicarbazide (FTSC) by incubating the RNA in freshly prepared fluorescent labeling solution (50 mM NaOAc, pH 5.3, 1.5 mM FTSC) at 37 °C for 1 h. The labeled RNA was ethanol precipitated and washed several times in cold ethanol prior to resuspension in 0.5x T.E. buffer and passaged over a G-25 column (GE Healthcare) to remove residual fluorophore. Purified FTSC-labeled RNA was stored at −20 °C until use. FAM-labeled DNA oligonucleotides were chemically synthesized (Integrated DNA Technologies). Prior to use, RNA was heated to 65 °C for one min and snap-cooled and DNA was annealed as described above. 3ʹ-FAM-labeled truncated RNA hairpins were purchased (Integrated DNA Technologies) and used without further purification.

### Fluorescence anisotropy

To determine the apparent binding affinities, appropriate concentrations of protein were incubated with either 2 nM DNA or 3 nM RNA in binding buffer (10 mM Tris-HEPES, pH 7.5, 8% Ficoll, 0.05% NP-40, 135 mM KCl, 15 mM NaCl, 1 mM DTT, 0.1 mg/mL non-acetylated BSA) for at least 1 h prior to FA measurements. Polarization was measured by a BMG Labtech CLARIOstar microplate reader using a 482 nm excitation filter with a 16 nm bandpass and a 530 nm emission filter with a 4 nm bandpass. Each titration was performed in triplicate and the polarization of each triplicate was measured three times to account for instrument error.

Competition and salt dependence binding assays were performed under the same FA conditions as described above with the following modifications. For competition using labeled DNA, 2 nM fluorescently labeled DNA and 5 nM Sox2-HMG were added separately to reactions containing serial dilutions of unlabeled nucleic acid competitor. Analogous experiments were performed with 3 nM fluorescently labeled RNA and 20 nM Sox2-HMG. The reactions equilibrated at room temperature overnight prior to measurement. The *K*_I_ was calculated from the IC_50_ by the Nikolovska–Coleska equation^72^. For salt dependence of select binding interactions, the monovalent salt concentration was varied but maintained a physiological 9:1 KCl:NaCl ratio. Binding curves were fit in Kaleidagraph using the following two-transition binding Eq. (1) that accounts for ligand depletion

1 $$A = A_0 + \left( {A_1 - A_0} \right) \ast \frac{{\left( {\left[ R \right]_{\mathrm{t}} \, + \, \left[ L \right]_{\mathrm{t}} \, + \, K_{{\mathrm{D}}1}} \right) - \sqrt {\left( {\left[ R \right]_{\mathrm{t}} \, + \, \left[ L \right]_{\mathrm{t}} + K_{{\mathrm{D}}1}} \right)^2 \, - \, 4\left[ R \right]_{\mathrm{t}}\left[ L \right]_{\mathrm{t}}} }}{{2\left[ R \right]_{\mathrm{t}}}} + \left( {A_2 - A_1} \right) \ast \left(\frac{{\left[ L \right]_{\mathrm{t}}}}{{\left[ L \right]_{\mathrm{t}} \,+ \, K_{{\mathrm{D}}2}}}\right)$$

where *A* = measured anisotropy, *A*_0_ = lower baseline, *A*_1_ = upper baseline of the first transition, [*R*]_t_ = total concentration of fluorescent probe, [*L*]_t_ = total concentration of protein, *K*_D1_ = dissociation constant of the first transition, *A*_2_ = upper baseline of the second transition, and *K*_D2_ = dissociation constant of the second transition. If a second transition was not detected, the fit was performed omitting the second binding term.

### Electrophoretic mobility shift assays

To validate select apparent binding affinities, binding reactions were assembled as in the fluorescent polarization experiments described above using trace DNA or RNA radiolabeled with ^32^P. Samples were electrophoresed on an 8% native polyacrylamide gel supplemented with 0.5x TBE buffer (45 mM Tris–HCI, 45 mM borate, pH 8.1, 1 mM EDTA) buffer and ran at 200 V for 30 min at room temperature. Gels were dried, imaged using a Typhoon PhosphoImager (Molecular Dynamics), and quantified by measuring the integrated signal density of the free and bound bands by the ImageQuant software suite. Data were fit in Kaleidagraph as a Langmuir binding isotherm. For stoichiometric EMSAs, the above reactions were assembled with either trace radiolabeled RNA or DNA supplemented with 2 µM RNA or DNA, respectively.

### Heteronuclear single quantum coherence NMR

^15^N-HSQC spectra were collected on an Oxford 600 MHz magnet equipped with an Agilent console and HCN PFG cold probe. Spectra were collected with ~500 µM ^15^N-labeled Sox2-HMG in NMR Buffer (10 mM NaH_2_PO_4_, pH 7.0, 10 mM DTT, 0.02% NaN_3_) as free protein or in fully-saturated 1:1.5 Sox2-HMG:nucleic acid complex with DNA or RNA at pH 6.5 and 25 °C. Spectra were analyzed via NMRPipe^73^ and visualized by CcpNmr Analysis^74^.

### Cell culture

Male mouse embryonic stem cells were maintained on 0.1% gelatin (EmbryoMax) coated plates in mESC media (KnockOut DMEM, Gibco), 12.6% fetal bovine serum (EmbryoMax), 1x MEM non-essential amino acids (Gibco), 1x GlutaMAX supplement (Gibco), Pen-Strep (100 U/mL penicillin and 100 mg/mL streptomycin, Gibco), 100 µM 2-mercaptoethanol (Gibco), 1000 U/mL LIF (ESGRO), 1 µM PD0325901 (Sigma-Aldrich), and 3 µM CHIR99021 (Sigma-Aldrich) to maintain pluripotency. Media was changed daily and cells were passaged every 2–3 days.

### Formaldehyde crosslinking

The fRIP protocol was adapted from a previous study^57^. Using 15-cm plates, mESC media was aspirated and the cells were washed once with 25 mL of RT 1x phosphate buffered saline (PBS) followed by submersion in 15 mL of room temperature PBS. Formaldehyde was added to 0.1%, allowed to crosslink for 10 min at room temperature, and quenched with the addition of glycine to 125 mM for 5 min. Cells were lifted, collected, and at 4 °C, spun down at 230 × *g* for 5 min. The pellet was washed twice with 10 mL of ice-cold PBS with centrifuge speeds of 230 × *g* 5 min for the first wash and 500 × *g* 5 min for the second wash, and followed by resuspension into 1.6 mL of ice-cold PBS. The suspension was centrifuged at 4000 × *g* for 75 s at 4 °C. The supernatant was removed, and the pellet was immediately flash frozen in liquid N_2_ and stored at −70 °C.

### UV crosslinking

Using 15-cm plates, mESC media was aspirated, replaced with 13 mL of ice-cold PBS, and subjected to 400 mJ/cm^2^ of 254 nm UV radiation (UV Stratalinker 1800). Cells were collected mechanically and at 4 °C, centrifuged at 500 × *g* for 5 min. The cell pellet was resuspended in 1 mL of ice-cold PBS and centrifuged at 4000 × *g* for 75 s at 4 °C. The supernatant was removed, and the pellet was immediately flash frozen in liquid N_2_ and stored at −70 °C.

### FRIP and UV-RIP

For fRIP and UV-RIP, cell pellets were resuspended in ice-cold RIPA lysis buffer (50 mM Tris, pH 8, 150 mM KCl, 0.1% SDS, 1% Triton X-100, 5 mM EDTA, 0.5% sodium deoxycholate, 0.5 mM DTT, 2x protease inhibitor cocktail (ThermoFisher), and 100 U/mL of RNAseOut (Invitrogen)), allowed to sit for 10 min, and sonicated (Bioruptor UCD-200) in an ice bath with high power setting and 30 s on/off for 10 min. At 4 °C the lysed cells were centrifuged at 16.1k × *g* for 10 min and the supernatant was collected. The number of cells collected after crosslinking for fRIP and UV-RIP were normalized to ~6.5 million cells. To preclear the supernatant for both fRIP and UV-RIP, 33 µL of Dynabeads Protein G (Invitrogen) and 550 µL of binding/washing buffer (25 mM Tris, pH 7.5, 150 mM KCl, 0.5% NP-40, 5 mM EDTA, 0.5 mM DTT, 1x PIC, and 100 U/mL RNAseOUT) was added and allowed to rotate for 45 min at 4 °C. From the pre-cleared supernatant, 50 µL was used as the input and 25 µL for a pre-Sox2 IP western blot with both being frozen at −20 °C. To the remaining supernatant, 5 µg of Sox2 antibody (Atlas Antibodies; validation shown in Supplementary Fig. 9) was added and rotated for 2 h at 4 °C. Next, 50 µL of Dynabeads protein G was added and allowed to rotate for 1 h at 4 °C. At this point, 25 µL of the supernatant was collected and frozen for a post-Sox2 IP western blot at −20 °C. The beads were washed twice with 1 mL of binding/washing buffer, the supernatant was removed, and the beads frozen at −20 °C.

### RNA purification and library preparation

At RT, to the input and beads, 83 µL of nuclease-free reverse-crosslinking buffer (1 × PBS, pH 7.4, 2% N-lauroyl sarcosine, 10 mM EDTA, 5 mM DTT, and 964 U/mL RNAseOUT) was added and allowed to mix well. From the resuspended bead sample, 3 µL was removed, frozen at −20 °C, and stored for a post-Sox2 IP western blot (Supplementary Fig. 9). To input and bead samples, 20 µL of proteinase K (New England Biolabs) was added. Samples were then heated in a thermocycler to 42 °C for 1 h and at 53 °C for another hour with gentle agitation to break apart settled beads. The input and bead samples were then vortexed with 1 mL of TRIzol Reagent (Ambion) followed by the addition of 200 µL of chloroform and 20 s of vigorous vortex. Samples were centrifuged at 16k × *g* for 10 min at 4 °C. The aqueous layer was transferred, mixed with 500 µL of isopropanol and 1 µL of GlycoBlue Coprecipitant (Invitrogen), and allowed to sit 10 min at 4 °C. Samples were centrifuged for 10 min at 12,000 × *g* at 4 °C, the supernatant was decanted, and the pellet allowed to air dry for 5 min. To input and bead samples, 85 µL of nuclease-free H_2_O, 10 µL of DNAse I reaction buffer, 1 µL of DNAse I (New England Biolabs) was added, and allowed to sit at room temperature for 15 min. RNA was cleaned up using RNeasy MinElute Cleanup Kit (Qiagen) following quick start protocol and eluted in RNAse free H_2_O and stored at −70 °C. The cDNA library was generated using KAPA RNA Hyperprep kit with RiboErase (HMR, Kapa Biosystems), 8 bp xGen dual index UMI adaptors (Integrated DNA Technologies), and sequenced on an Illumina NextSeq 500 using paired end sequencing of 34 bp.

### Computational pipeline for fRIP- and UV-RIP-seq

Sequencing reads were mapped by STAR^75^ to mm38 using a gene annotation from GENCODE (vM16)^76^. The reads that are not uniquely mapped were discarded. The unique molecular identifiers (UMIs) associated with sequencing reads were counted for gene bodies in the annotation by using featureCounts^77^. The enriched genes were identified by comparing pull-down with inputs. The fold changes of pull-down relative to input and their statistical significance were calculated by DESeq2^78^ for the genes that have three or more UMIs in at least one sample. Specifically, the total number of UMIs of uniquely mapped reads were used as a normalization factor, and biological replicate information were considered as a covariate.

### Reporting summary

Further information on research design is available in the Nature Research Reporting Summary linked to this article.

## Supplementary information

Supplementary Information

Peer Review

Reporting Summary

## Data Availability

Computation code available upon request.

## Source data

Source Data

## Data availability

The data that support this study are available upon reasonable request. All sequencing data generated have been deposited in the NCBI GEO database (https://www.ncbi.nlm.nih.gov/gds/) under accession code GSE142013. The source data for Figs. 1, 2c, 3, 4, 6, Supplementary Figs. 4, 5, 8, and 9, and Supplementary Tables 1 and 7 are provided as a source data file.

## References

[1] Cassiday LA, Maher LJ (2002). Having it both ways: transcription factors that bind DNA and RNA. Nucleic Acids Res..

[2] Hudson WH, Ortlund EA (2014). The structure, function and evolution of proteins that bind DNA and RNA. Nat. Rev. Mol. Cell Biol..

[3] Hung T (2011). Extensive and coordinated transcription of noncoding RNAs within cell-cycle promoters. Nat. Genet..

[4] Sigova AA (2015). Transcription factor trapping by RNA in gene regulatory elements. Science.

[5] Tournillon, A. S. et al. p53 binds the mdmx mRNA and controls its translation. *Oncogene* **36**, 723–730 (2017). 10.1038/onc.2016.236 27375027

[6] Yoshida, Y. et al. Binding of RNA to p53 regulates its oligomerization and DNA-binding activity. *Oncogene* **23**, 4371–4379 (2004). 10.1038/sj.onc.1207583 15064727

[7] Shamovsky I, Ivannikov M, Kandel ES, Gershon D, Nudler E (2006). RNA-mediated response to heat shock in mammalian cells. Nature.

[8] Baltz AG (2012). The mRNA-bound proteome and its global occupancy profile on protein-coding transcripts. Mol. Cell.

[9] Castello A (2012). Insights into RNA biology from an atlas of mammalian mRNA-binding proteins. Cell.

[10] Kwon SC (2013). The RNA-binding protein repertoire of embryonic stem cells. Nat. Struct. Mol. Biol..

[11] Castello A (2016). Comprehensive identification of RNA-binding domains in human cells. Mol. Cell.

[12] Conrad, T. et al. Serial interactome capture of the human cell nucleus. *Nat. Commun.*10.1038/ncomms11212 (2016). 10.1038/ncomms11212 PMC4822031 27040163

[13] He C (2016). High-resolution mapping of RNA-binding regions in the nuclear proteome of embryonic stem cells. Mol. Cell.

[14] Mullari M, Lyon D, Jensen LJ, Nielsen ML (2017). Specifying RNA-binding regions in proteins by peptide cross-linking and affinity purification. J. Proteome Res..

[15] Bao X (2018). Capturing the interactome of newly transcribed RNA. Nat. Methods.

[16] Moller K, Rinke J, Ross A, Buddle G, Brimacombe R (1977). The use of formaldehyde in RNA-protein cross-linking studies with ribosomal subunits from *Escherichia coli*. Eur. J. Biochem..

[17] Sarlak G, Vincent B (2016). The roles of the stem cell-controlling Sox2 transcription factor: from neuroectoderm development to Alzheimer’s disease?. Mol. Neurobiol..

[18] Rizzino A, Wuebben EL (2016). Sox2/Oct4: a delicately balanced partnership in pluripotent stem cells and embryogenesis. Biochim. Biophys. Acta.

[19] Suwinska A, Czolowska R, Ozdzenski W, Tarkowski AK (2008). Blastomeres of the mouse embryo lose totipotency after the fifth cleavage division: expression of Cdx2 and Oct4 and developmental potential of inner and outer blastomeres of 16- and 32-cell embryos. Dev. Biol..

[20] Chew JL (2005). Reciprocal transcriptional regulation of Pou5f1 and Sox2 via the Oct4/Sox2 complex in embryonic stem cells. Mol. Cell Biol..

[21] Avilion AA (2003). Multipotent cell lineages in early mouse development depend on SOX2 function. Genes Dev..

[22] Boer B (2007). Elevating the levels of Sox2 in embryonal carcinoma cells and embryonic stem cells inhibits the expression of Sox2:Oct-3/4 target genes. Nucleic Acids Res..

[23] Kopp JL, Ormsbee BD, Desler M, Rizzino A (2008). Small increases in the level of Sox2 trigger the differentiation of mouse embryonic stem cells. Stem Cells.

[24] Takahashi K, Yamanaka S (2006). Induction of pluripotent stem cells from mouse embryonic and adult fibroblast cultures by defined factors. Cell.

[25] Yu J (2007). Induced pluripotent stem cell lines derived from human somatic cells. Science.

[26] Huangfu D (2008). Induction of pluripotent stem cells from primary human fibroblasts with only Oct4 and Sox2. Nat. Biotechnol..

[27] Maucksch C, Jones KS, Connor B (2013). Concise review: the involvement of SOX2 in direct reprogramming of induced neural stem/precursor cells. Stem Cell Transl. Med..

[28] Feng R, Wen J (2015). Overview of the roles of Sox2 in stem cell and development. Biol. Chem..

[29] Bylund M, Andersson E, Novitch BG, Muhr J (2003). Vertebrate neurogenesis is counteracted by Sox1-3 activity. Nat. Neurosci..

[30] Guo X (2018). LincRNA-1614 coordinates Sox2/PRC2-mediated repression of developmental genes in pluripotency maintenance. J. Mol. Cell Biol..

[31] Ng SY, Johnson R, Stanton LW (2012). Human long non-coding RNAs promote pluripotency and neuronal differentiation by association with chromatin modifiers and transcription factors. EMBO J..

[32] Ng SY, Bogu GK, Soh BS, Stanton LW (2013). The long noncoding RNA RMST interacts with SOX2 to regulate neurogenesis. Mol. Cell.

[33] Samudyata (2019). Interaction of Sox2 with RNA binding proteins in mouse embryonic stem cells. Exp. Cell Res..

[34] Mallam AL (2019). Systematic discovery of endogenous human ribonucleoprotein complexes. Cell Rep..

[35] Lee MS (2016). SOX2 suppresses the mobility of urothelial carcinoma by promoting the expression of S100A14. BB Rep..

[36] Yuan H, Corbi N, Basilico C, Dailey L (1995). Developmental-specific activity of the FGF-4 enhancer requires the synergistic action of Sox2 and Oct-3. Genes Dev..

[37] Soufi A (2015). Pioneer transcription factors target partial DNA motifs on nucleosomes to initiate reprogramming. Cell.

[38] Moosa, M. M., Tsoi, P. S., Choi, K. J., Ferreon, A. C. M. & Ferreon, J. C. Direct single-molecule observation of sequential DNA bending transitions by the Sox2 HMG Box. *Int. J. Mol. Sci.*10.3390/ijms19123865 (2018). 10.3390/ijms19123865 PMC6321608 30518054

[39] Scaffidi P, Bianchi ME (2001). Spatially precise DNA bending is an essential activity of the sox2 transcription factor. J. Biol. Chem..

[40] Polaski JT, Holmstrom ED, Nesbitt DJ, Batey RT (2016). Mechanistic insights into cofactor-dependent coupling of RNA folding and mRNA transcription/translation by a cobalamin riboswitch. Cell Rep..

[41] Hou L, Srivastava Y, Jauch R (2017). Molecular basis for the genome engagement by Sox proteins. Semin Cell Dev. Biol..

[42] Hagey DW, Muhr J (2014). Sox2 acts in a dose-dependent fashion to regulate proliferation of cortical progenitors. Cell Rep..

[43] Chakravarthy H (2008). Identification of DPPA4 and other genes as putative Sox2:Oct-3/4 target genes using a combination of in silico analysis and transcription-based assays. J. Cell Physiol..

[44] Ding Y, Chan CY, Lawrence CE (2004). Sfold web server for statistical folding and rational design of nucleic acids. Nucleic Acids Res..

[45] Remenyi A (2003). Crystal structure of a POU/HMG/DNA ternary complex suggests differential assembly of Oct4 and Sox2 on two enhancers. Genes Dev..

[46] Williams DJ, Cai M, Clore G (2004). Molecular basis for synergistic transcriptional activation by Oct1 and Sox2 revealed from the solution structure of the 42-kDa Oct1·Sox2·Hoxb1-DNA ternary transcription factor complex. J. Biol. Chem..

[47] Record MT, Lohman ML, De Haseth P (1976). Ion effects on ligand-nucleic acid interactions. J. Mol. Biol..

[48] Privalov PL, Dragan AI, Crane-Robinson C (2011). Interpreting protein/DNA interactions: distinguishing specific from non-specific and electrostatic from non-electrostatic components. Nucleic Acids Res..

[49] Love JJ, Li X, Chung J, Dyson HJ, Wright PE (2004). The LEF-1 high-mobility group domain undergoes a disorder-to-order transition upon formation of a complex with cognate DNA. Biochemistry.

[50] Crane-Robinson C (1998). The energetics of HMG box interactions with DNA. Thermodynamic description of the box from mouse Sox-5. J. Mol. Biol..

[51] van Houte LP (1995). Solution structure of the sequence-specific HMG box of the lymphocyte transcriptional activator Sox-4. J. Biol. Chem..

[52] Luscombe NM, Laskowski RA, Thornton JM (2001). Amino acid-base interactions: a three-dimensional analysis of protein-DNA interactions at an atomic level. Nucleic Acids Res..

[53] Stros M, Launholt D, Grasser KD (2007). The HMG-box: a versatile protein domain occurring in a wide variety of DNA-binding proteins. Cell Mol. Life Sci..

[54] Schaukowitch K, Joo JY, Kim TK (2017). UV-RNA immunoprecipitation (UV-RIP) protocol in neurons. Methods Mol. Biol..

[55] Islam S (2014). Quantitative single-cell RNA-seq with unique molecular identifiers. Nat. Methods.

[56] Sena, J. A. et al. Unique Molecular Identifiers reveal a novel sequencing artefact with implications for RNA-Seq based gene expression analysis. *Sci. Rep.*10.1038/s41598-018-31064-7 (2018). 10.1038/s41598-018-31064-7 PMC6120941 30177820

[57] Hendrickson, D. G. et al. binding by chromatin-associated proteins. *Genome Biol.*10.1186/s13059-016-0878-3 (2016). 10.1186/s13059-016-0878-3 PMC4756407 26883116

[58] Werner MH, Huth JR, Gronenborn AM, Clore GM (1995). Molecular basis of human 46X,Y sex reversal revealed from the three-dimensional solution structure of the human SRY-DNA complex. Cell.

[59] Bewley CA, Gronenborn AM, Clore GM (1998). Minor groove-binding architectural proteins: structure, function, and DNA recognition. Annu Rev. Bioph Biom..

[60] Parvin JD, McCormick RJ, Sharp PA, Fisher DE (1995). Pre-bending of a promoter sequence enhances affinity for the TATA-binding factor. Nature.

[61] Weiss MA (2001). Floppy SOX: mutual induced fit in HMG (high-mobility group) box-DNA recognition. Mol. Endocrinol..

[62] Liu ZR, Wilkie AM, Clemens MJ, Smith CW (1996). Detection of double-stranded RNA-protein interactions by methylene blue-mediated photo-crosslinking. RNA.

[63] Ng SY, Lin L, Soh BS, Stanton LW (2013). Long noncoding RNAs in development and disease of the central nervous system. Trends Genet..

[64] Fang X (2011). Landscape of the SOX2 protein-protein interactome. Proteomics.

[65] Gao Z (2012). Determination of protein interactome of transcription factor Sox2 in embryonic stem cells engineered for inducible expression of four reprogramming factors. J. Biol. Chem..

[66] Mallanna SK (2010). Proteomic analysis of Sox2-associated proteins during early stages of mouse embryonic stem cell differentiation identifies Sox21 as a novel regulator of stem cell fate. Stem Cells.

[67] Tung CL (2010). SOX2 modulates alternative splicing in transitional cell carcinoma. Biochem Biophys. Res. Commun..

[68] Sambrook, J., Fritsch, E. F. & Maniatis, T. *Molecular Cloning: A Laboratory Manual*, 2nd edn (Cold Spring Harbor Press, 1989).

[69] Ho SN, Hunt HD, Horton RM, Pullen JK, Pease LR (1989). Site-directed mutagenesis by overlap extension using the polymerase chain reaction. Gene.

[70] Edwards AL, Garst AD, Batey RT (2009). Determining structures of RNA aptamers and riboswitches by X-ray crystallography. Methods Mol. Biol..

[71] Zearfoss NR, Ryder SP (2012). End-labeling oligonucleotides with chemical tags after synthesis. Methods Mol. Biol..

[72] Nikolovska-Coleska Z (2004). Development and optimization of a binding assay for the XIAP BIR3 domain using fluorescence polarization. Anal. Biochem..

[73] Delaglio F (1995). NMRPipe: a multidimensional spectral processing system based on UNIX pipes. J. Biomol. NMR.

[74] Vranken WF (2005). The CCPN data model for NMR spectroscopy: development of a software pipeline. Proteins.

[75] Dobin A (2013). STAR: ultrafast universal RNA-seq aligner. Bioinformatics.

[76] Frankish A (2019). GENCODE reference annotation for the human and mouse genomes. Nucleic Acids Res..

[77] Liao Y, Smyth GK, Shi W (2014). featureCounts: an efficient general purpose program for assigning sequence reads to genomic features. Bioinformatics.

[78] Love, M. I., Huber, W. & Anders, S. Moderated estimation of fold change and dispersion for RNA-seq data with DESeq2. *Genome Biol.*10.1186/s13059-014-0550-8 (2014). 10.1186/s13059-014-0550-8 PMC4302049 25516281

