# Supplementary material for: The Sox2 transcription factor binds RNA
Source: Nat Commun. 2020 Apr 14;11:1805. doi: 10.1038/s41467-020-15571-8 (PMC7156710; doi:10.1038/s41467-020-15571-8)
Supplement: Supplementary file 1 — Supplementary Information [file 41467_2020_15571_MOESM1_ESM.pdf]

The Sox2 transcription factor binds RNA

Zachariah E. Holmes[1,‡], Desmond J. Hamilton[1,‡], Taeyoung Hwang[1], Nicholas V. Parsonnet[1], John L. Rinn[1,2], Deborah S. Wuttke[1,*] and Robert T. Batey[1,*]

[1]Department of Biochemistry, [2]BioFrontiers Institute, University of Colorado at Boulder, Campus Box 596, Boulder, Colorado, USA, 80309-0596

[‡]These authors made equal contributions.

*Corresponding authors: deborah.wuttke@colorado.edu, robert.batey@colorado.edu

SUPPLEMENTARY INFORMATION.

Enhancer element	(+) Strand Sequence	$K_{D,app}$ (nM)
FGF4	CGCGC**CTTTGTT**CCCGGGT	0.9 ± 0.2
SOX2	CGCGC**CATTGTG**CCCGGGT	0.9 ± 0.2
DPPA4	CGCGC**ATTTGTA**CCCGGGT	3.4 ± 1.0
CCND1_SM1	CGCGC**CCTCTTC**CCCGGGT	9.8 ± 1.1
CCND1_SM6	CGCGC**ACTACAG**CCCGGGT	24 ± 5

Supplementary Table 1. Sox2-HMG binds dsDNA targets with a range of affinities. Binding affinities were measured for a set of *in vivo* Sox2 DNA targets from the enhancer regions of the indicated genes and placed in the context of a model duplex. The apparent binding affinity ($K_{D,app}$) is presented as the average of n=3 replicates and the standard error of the mean (s.e.m.).

Supplementary Table 2. Sequences of RNA ligands used in this study.

RNA	Length (nt)	Sequence (5' – 3')
ES2 lncRNA	354	GGAAGAAGUGAAAUCACUAUAUUUAGAAACGAAUGCUACC AUCCCACCCCAACUACAUGUUCAGGUUAAAUGCGUGCACA UCCUGGUAGCUGGAACAUCUGUUUCUAGCUUGUUGUUCCU CUUGGCUGCUUUUCAAUUGGAUUUCCUCGAGCAGGUCCCA GGCUUGUCACUGCAGCCAGGGUCUGUUAACCAUCACCACU GGUUCUACAUUAAGUAGGAGGUUCAGGGCUAAGCUAAUAA CUCGCCUGCCUUUCUGACUGAUCACAGUGAUGUUCGCCGU GGCCACUUAUCAUGCGGGCUCCUUUGGGAGGGUGCUCUUC UUUGUCUGUACCUCUGGGUGGAAAGGGCGAAUUC
env8 ssRNA	36	AUACAACAUACAACAUACAACAUACAACAUACAACA
ES2, nts 1-178	178	GGAAGAAGUGAAAUCACUAUAUUUAGAAACGAAUGCUACC AUCCCACCCCAACUACAUGUUCAGGUUAAAUGCGUGCACA UCCUGGUAGCUGGAACAUCUGUUUCUAGCUUGUUGUUCCU CUUGGCUGCUUUUCAAUUGGAUUUCCUCGAGCAGGUCCCA GGCUUGUCACUGCAGCCA
ES2, nts 89-267	180	GGCUGGAACAUCUGUUUCUAGCUUGUUGUUCCUCUUGGCU GCUUUUCAAUUGGAUUUCCUCGAGCAGGUCCCAGGCUUGU CACUGCAGCCAGGGUCUGUUAACCAUCACCACUGGUUCUA CAUUAAGUAGGAGGUUCAGGGCUAAGCUAAUAACUCGCCU GCCUUUCUGACUGAUCACAG
ES2, nts 179-354	176	GGGUCUGUUAACCAUCACCACUGGUUCUACAUUAAGUAGG AGGUUCAGGGCUAAGCUAAUAACUCGCCUGCCUUUCUGAC UGAUCACAGUGAUGUUCGCCGUGGCCACUUAUCAUGCGGG CUCCUUUGGGAGGGUGCUCUUCUUUGUCUGUACCUCUGGG UGGAAAGGGCGAAUUC
ES2, nts 1-89	89	GGAAGAAGUGAAAUCACUAUAUUUAGAAACGAAUGCUACC AUCCCACCCCAACUACAUGUUCAGGUUAAAUGCGUGCACA UCCUGGUAG
ES2, nts 89-178	91	GGCUGGAACAUCUGUUUCUAGCUUGUUGUUCCUCUUGGCU GCUUUUCAAUUGGAUUUCCUCGAGCAGGUCCCAGGCUUGU CACUGCAGCCA
ES2, nts 179-267	89	GGGUCUGUUAACCAUCACCACUGGUUCUACAUUAAGUAGG AGGUUCAGGGCUAAGCUAAUAACUCGCCUGCCUUUCUGAC UGAUCACAG

ES2, nts 267-354	89	GGUGAUGUUCGCCGUGGCCACUUAUCAUGCGGGCUCCUUUGGGAGGGUGCUCUUCUUUGUCUGUACCUCUGGGUGGAAAGGGCGAAUUC
Loop A	40	GGUCGCCGUGGCCACUUCGAAAGGGGUGGAAAGGGCGACC
Loop B	39	GGUCUUAUCAUGCGGGCGAAAGUCUGUACCUCUGGGACC
Loop C	38	GGUGGGCUCCUUUGGGAGGGUGCUCUUCUUUGUCUACC
Loop B, P1(5bp)	35	GGUUAUCAUGCGGGCGAAAGUCUGUACCUCUGGCC
Loop B, P1(3bp)	31	GGAUCAUGCGGGCGAAAGUCUGUACCUCUCC
Loop B, P2(5bp)	35	GGUCUUAUCAUGCGCGAAAGUGUACCUCUGGGACC
Loop B, P2(3bp)	31	GGUCUUAUCAUGCGAAAGUACCUCUGGGACC
Loop B, P1/2(3bp)	27	GGUAUCAUGCGGAAACGCACCUCUACC
Loop B, Bulge(2+3)	39	GGUCUUAUCGUGCGGGCGAAAGUCUGUACCUCUGGGACC
Loop B, Bulge(1+2)	39	GGUCUUAUGGUGCGGGCGAAAGUCUGUACCUCUGGGACC
Loop B, Bulge(0+1)	39	GGUCUUAAGGUGCGGGCGAAAGUCUGUACCUCUGGGACC
Loop B, Fully Paired	38	GGUCUUAUCAUGCGGGCGAAAGUCUGUAUGAUGGGACC
Loop B, Duplex_Strand A	17	GGUCUUAUCAUGCGGGC
Loop B, Duplex_Strand B	17	GCCCGCAUGAUAAGACC
xpt-pbuX	67	GGACAUAUAAUCGCGUGGAUAUGGCACGCAAGUUUCUACCGGGCACCGUAAAUGUCCGACUAUGUCC
env4	103	GGUGUUAAAAGCAUAGUGGGAAAGUGACGUGUAAUUCGUCCACAUUACUUGAUGCGGUUAUAGUCCCAAUGCCACCUAACACAAAGUACGAGCAAGGAGACUC
$tRNA^{Leu(CAG)}$	93	GCGAAGGUGGCGGAAUUGGUAGACGCGCUAGCUUCAGGUGUUAGUGUCCUUACGGACGUGGGGGUUCAAGUCCCCCCCCUCGCACCAGGAUCU
13 bp Hairpin	30	GGUCCAUGCGGGCGAAAGUCUGUAUGGACC

9 bp Hairpin	22	GGUCCAUGCGAAAGUAUGGACC
5 bp Hairpin	14	GGUCCGAAAGGACC

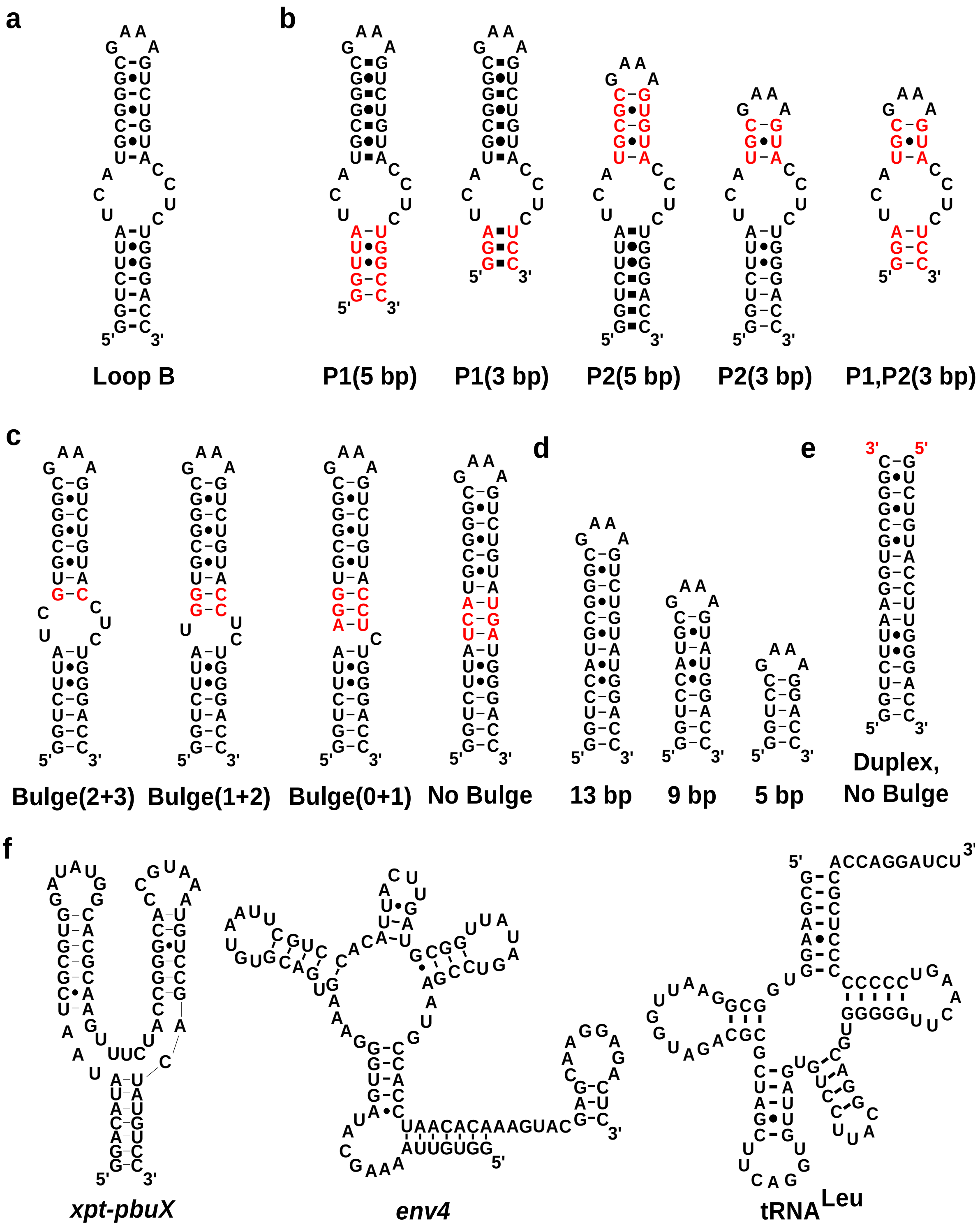

Supplementary Figure 3. Secondary structures of RNAs used in this study. **a** Loop B hairpin in which mutations were made. **b** Helix length mutants. **c** Internal loop mutants. **d** Truncated hairpins. **e** Terminal loop mutant. **f** Highly structured RNAs.

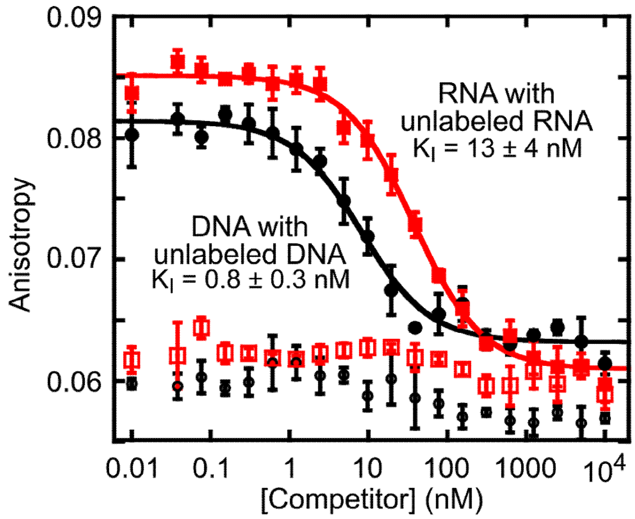

Supplementary Figure 4. Self-competition experiments were performed between fluorescently labeled and unlabeled nucleic acid either in the presence (closed shapes) or absence (open shapes) of Sox2-HMG. Binding curves presented as the average of 3 technical replicates with the error bars reflecting the standard deviation.

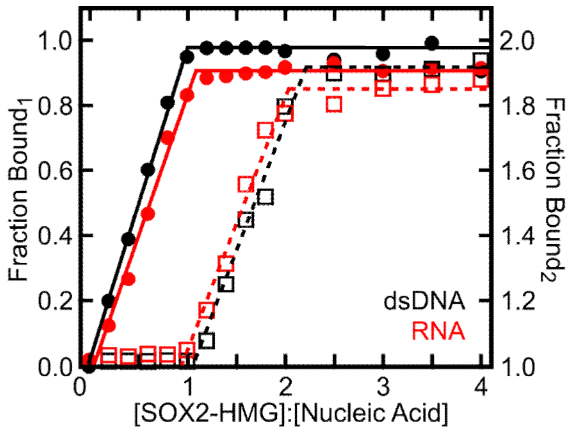

Supplementary Figure 5. Quantification of stoichiometric EMSAs. The calculated fraction bound for dsDNA (black) and RNA (red) are plotted as a function of the [Sox2-HMG]:[Nucleic Acid] with the first transition indicated in closed circles and the second in open squares.

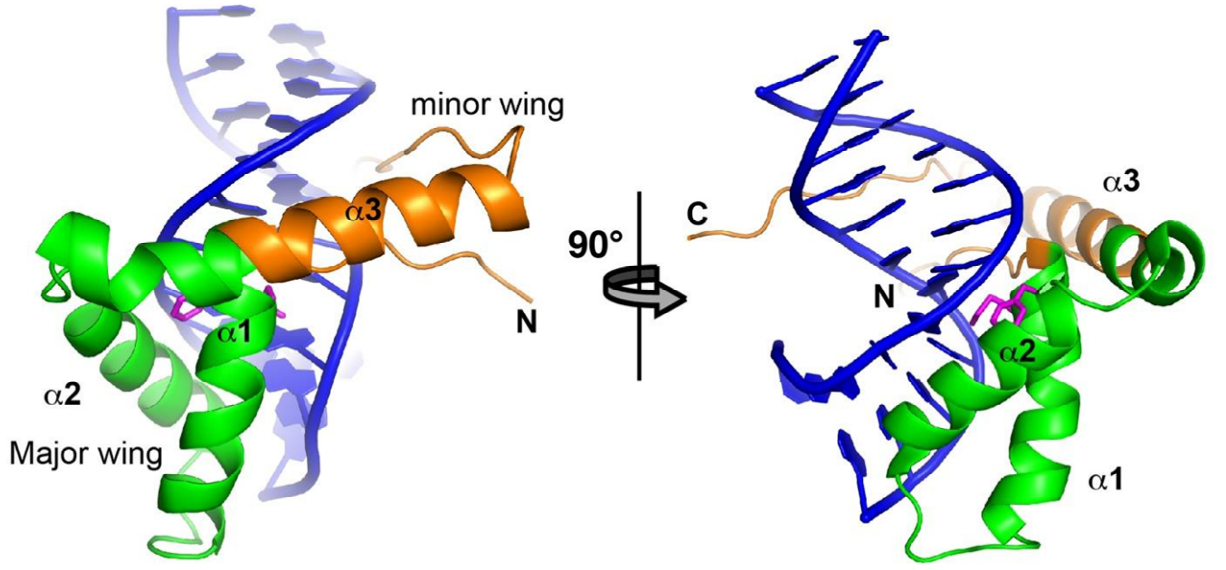

Supplementary Figure 6. Architecture of the Sox2-DNA interaction (PDB ID 1GT0). The high mobility group (HMG) domain of Sox2 is divided into two subdomains: the major wing (green) and minor wing (orange). Interactions occur with the DNA (blue) through the minor groove; two amino acids (F48 and M49, magenta) form a hydrophobic wedge that facilitates a ~90° bend in the DNA towards the major groove.

Supplementary Table 7.

	DNA		RNA			
	$K_{D,rel}$	SD	$K_{D,rel}$	SD	P-value	$K_{D,rel}$ DNA / $K_{D,rel}$ RNA
R40	2.0	1.9	2.7	0.3	5.8E-01	0.74
K42	2.8	1.8	7.9	2.7	6.0E-02	0.35
R43	8.5	2.2	2.8	0.6	3.6E-02	**3.0**
M45	1.4	0.7	0.9	0.5	4.0E-01	1.6
N46	15	8.4	3.5	1.1	1.4E-01	**4.3**
F48	420	120	16	2	2.9E-02	**26**
M49	30	5.4	0.7	0.4	1.1E-02	**43**
W51	17	2.8	78	13	1.3E-02	**0.21**
R53	0.7	0.2	1.7	0.6	9.8E-02	0.39
R56	190	70	3.0	0.3	4.5E-02	**63**
R57	0.8	0.3	1.3	0.5	3.0E-01	0.64
K58	1.1	0.5	0.8	0.4	4.5E-01	1.4
Q61	0.6	0.2	0.8	0.5	6.3E-01	0.73
E62	0.6	0.3	1.6	0.8	1.5E-01	0.36
N63	2.9	1.4	1.3	0.2	2.0E-01	2.2
P64	2.6	0.1	2.0	0.7	3.0E-01	1.3
K65	8.5	1.0	2.6	0.7	1.5E-03	**3.3**
M66	1.8	0.9	1.3	0.5	4.6E-01	1.3
H67	2.4	0.7	3.7	1.7	3.1E-01	0.65
N68	1.1	0.5	1.0	0.1	8.1E-01	1.1
S69	1.1	0.5	1.6	0.5	3.2E-01	0.69
E70	0.7	0.4	1.2	0.4	2.0E-01	0.56
S72	1.4	0.4	1.0	0.6	4.0E-01	1.4
K73	3.0	0.6	6.9	0.2	3.8E-03	0.43
R74	0.7	0.4	0.9	0.5	5.9E-01	0.74
E78	2.2	1.0	0.9	0.5	1.3E-01	2.4
W79	490	20	50	19	1.4E-05	**9.9**
K80	2.8	0.9	40	15	5.4E-02	**0.07**
S83	0.9	0.2	1.8	0.6	9.0E-02	0.50
E84	1.6	0.3	0.9	0.5	9.0E-02	1.8
T85	2.8	0.3	0.9	0.3	8.0E-04	**3.1**
E86	0.8	0.1	0.9	0.6	8.4E-01	0.93
K87	9.3	1.4	4.1	1.4	9.7E-03	2.3
R88	1.3	0.7	0.7	0.2	3.0E-01	1.8
K95	1.1	1.1	3.2	1.2	8.8E-02	0.34
R96	1.6	1.1	0.8	0.3	3.4E-01	2.0
R98	0.8	0.5	5.5	1.9	4.4E-02	**0.15**
H101	1.9	0.2	2.2	0.9	5.3E-01	0.84

K103	1.4	1.3	2.5	1.1	3.3E-01	0.56
E104	1.1	0.3	1.0	0.5	7.5E-01	1.1
H105	1.7	1.9	2.0	1.0	8.0E-01	0.85
K109	3.2	0.8	7.4	2.5	9.0E-02	0.43
Y110	7.5	3.5	1.7	0.6	9.6E-02	**4.4**
R111	3.0	0.8	2.2	1.2	3.6E-01	1.4
P112	2.7	1.2	1.8	0.8	3.1E-01	1.5
R113	0.6	0.5	3.1	1.3	6.9E-02	**0.19**
R114	6.1	2.2	9.2	2.3	1.7E-01	0.66
K115	5.8	1.0	3.6	1.4	9.1E-01	1.6

Supplementary Table 7. Measured $K_{D,rel}$(DNA) and $K_{D,rel}$(RNA) for each alanine point mutant. The values of $K_{D,rel}$ are presented as the average and standard deviation of 3 technical replicates. Values are colored according to the scale in Fig.6. P-values for the difference between $K_{D,rel}$(DNA) and $K_{D,rel}$(DNA) were calculated by the student's t-test assuming non-parametric distribution.

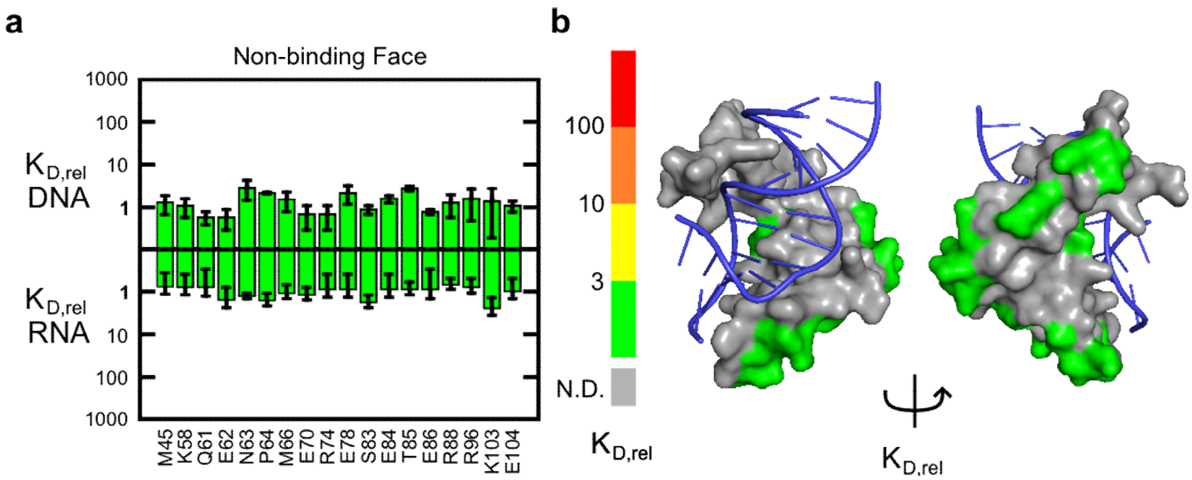

Supplementary Figure 8. Effect of Sox2-HMG non-binding surface alanine point mutations on nucleic acid binding. **a** The values of $K_{D,rel}$ for DNA and RNA binding are presented as the average and standard deviation of 3 technical replicates. **b** $K_{D,rel}$ for both DNA and RNA binding mapped to the surface of Sox2-HMG complex (PDB ID 1GT0) using the color scale provided.

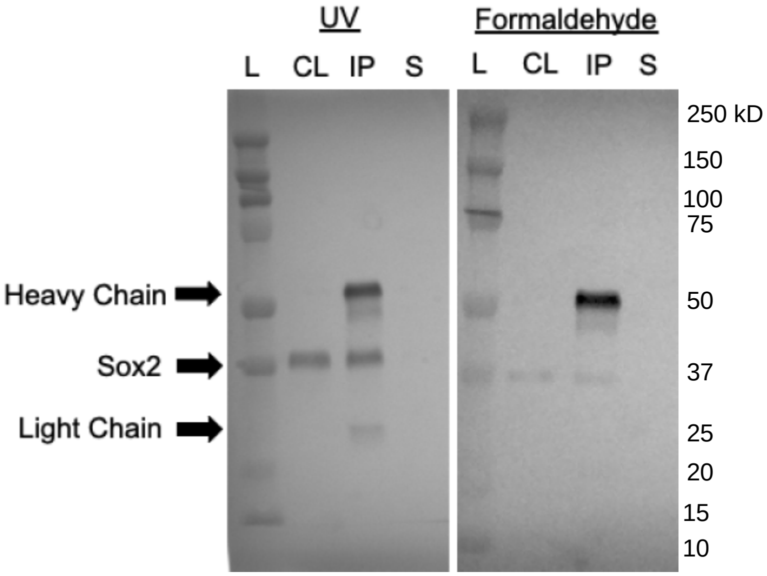

Supplementary Figure 9. UVRIP and fRIP immunoprecipitates Sox2. Western blots, using the same antibody as in the UVRIP and fRIP assays, show that Sox2 antibody is specific for Sox2 in crosslinked mESC whole cell lysate (**CL**), Sox2 is pulled down with immunoprecipitation (**IP**), and Sox2 is not detected in the supernatant (**S**) post IP. Ladder (**L**).

Supplementary Table 10. Sequences of proteins used in this study.

Protein	Sequence
Sox2	HHHHHHSSGLVPRGSHMASMYNMMETELKPPGPQQTSGGGGGNSTAAAAGGNQKNSPD RVKRPMNAFMVWSRGQRRKMAQENPKMHNSEISKRLGAEWKLLSETEKRPFIDEAKRL RALHMKEHPDYKYRPRRKTKTLMKKDKYTLPGGLLAPGGNSMASGVGVGAGLGAGVNQ RMDSYAHMNGWSNGSYSMMQDQLGYPQHPGLNAHGAAQMQPMHRYDVSALQYNSMTSS QTYMNGSPTYSMSYSQQGTPGMALGSMGSVVKSEASSSPPVVTSSSHSRAPCQAGDLR DMISMYLPGAEVPEPAAPSRLHMSQHYQSGPVPGTAINGTLPLSHM
Sox2-HMG	MGSSHHHHHHSSGLVPRGSHMPDRVKRPMNAFMVWSRGQRRKMAQENPKMHNSEISKR LGAEWKLLSETEKRPFIDEAKRLRALHMKEHPDYKYRPRRKTKTLMK
Sox2-HMG (Thrombin cleaved)	GSHMPDRVKRPMNAFMVWSRGQRRKMAQENPKMHNSEISKRLGAEWKLLSETEKRPFI DEAKRLRALHMKEHPDYKYRPRRKTKTLMK
Sox2-HMG (HRV 3C Protease cleaved)	GPHMPDRVKRPMNAFMVWSRGQRRKMAQENPKMHNSEISKRLGAEWKLLSETEKRPFI DEAKRLRALHMKEHPDYKYRPRRKTKTLMK
