# Supplementary material for: The Sox2 transcription factor binds RNA
Source: Nat Commun. 2020 Apr 14;11:1805. doi: 10.1038/s41467-020-15571-8 (PMC7156710; doi:10.1038/s41467-020-15571-8)
Supplement: Supplementary file 2 — Peer Review [file 41467_2020_15571_MOESM2_ESM.pdf]

Reviewers' comments:

Reviewer #1 (Remarks to the Author):

The authors make a compelling biochemical case for the ability of the Sox2 protein to bind double stranded RNA, in addition to its normal double stranded DNA target. This is an important observation, since Sox2 is normally thought of as a transcription factor, such that effect it exerts in cells would be attributed to that function. Certainly I am convinced that Sox2 can associate with double-stranded RNA, but I'm left with many questions regarding the optimal RNA target.

Additional details about HMG-box proteins would put these results in perspective. There appears to be a consensus that HMG-box proteins prefer to bind to non-canonical B-form DNAs. In particular, they bind to kinked or unwound duplex DNA (noting, however, that the crystal structure 1GT0 used in Figure 6 contains a normal dsDNA, although Sox2 is bound at the blunt end of the duplex which looks as if it is unwound). Also, they are known to introduce a bend in duplex DNA. With these details in mind, the ability of Sox2 to bind dsRNA is still surprising, but of all DNA binding proteins, HMG-boxes might be the most likely to bind.

The authors assume that the reader is familiar with the architecture of HMG boxes. For example, they refer to the major and minor wings of the protein, but they do not show a protein structure and identify these elements. The structures shown in Figure 6 from the cocrystals structure do not help the reader, since this surface rendering obscures the juxtaposition of the α-helices and the position of the tails. Please provide another figure that explicitly shows the protein's elements.

Questions about RNA targets and relative binding affinities:
1. Sox2 seems to prefer an RNA duplex with an internal bulge, rather than a perfect duplex (Figure 3c). The nucleic acid targets for Sox2 in Figure 3 need more descriptions: why, for example, does Sox2 in 3c,d bind to the hairpin with a perfect duplex (17 base pairs) with KD=15 nM, but in 3f to an RNA duplex with KD,app = 35 nM? (The authors explain results in 3d as due to poor annealing of the two RNA strands). The authors suggest (ll 152-155) that other structural features could contribute to Sox2 binding, which is supported by their data, but also agrees with what is known about the imperfect dsDNAs that Sox2 prefers. More emphasis on this preference and how it translates to the typically short and imperfect RNA duplexes in RNA structures could be made.

2. What RNAs were used in S4 and S5? Which nucleic acids were added to Sox2 HMG in the NMR experiments?

3. Do the authors have any ideas about the RNA binding site size for Sox2? I imagine that in the RNA hairpins with an internal bulge, Sox2 is sitting on the duplex between hairpin loop and bulge, to maximize its contact with a deformed A-form duplex.

4. A concern in Figure 2c: they monitor the 3'fluorophore during binding, and fit to a 1:1 binding isotherm. The concentration of RNA (3 nM) is very close to their measured KD; did they repeat these experiments at lower RNA concentrations? Did they measure stoichiometry?

5. A peculiarity: Competition assays are described (ll 474-475) as equilibrating overnight. Is overnight at room temp necessary? If so, how do the authors explain this? Conformational changes of nucleic acids or Sox2? Incredibly slow off-rates (not consistent with the KD)?

6. Figure 6a with the DNA duplex bound is from the cocrystals. Have the authors tried to model in an A-form duplex? Not easy to do, but it could be informative.

7. Figure 5 is quite striking – is there any reason to expect that the tails of the protein are not involved with RNA binding?

Reviewer #2 (Remarks to the Author):

This manuscript reports the results of a study on the transcription factor Sox2. The main focus of the work is the characterization of the interaction of Sox2 with RNA. The authors observe that Sox2 directly binds to RNA using its high mobility group (HMG) DNA-binding domain. In addition, they report that RNA recognition occurs through binding to double stranded RNA (dsRNA) in a non-specific fashion. These results indicate that interaction of Sox2 with RNA may play an important role in how Sox2 regulates gene expression.

This manuscript is an interesting and rigorous study that provides substantial new information to understand a novel and, so far, not well characterized interaction of Sox2 with RNA that is potentially very important for its function. For these reasons I believe it will be interesting to the readers of Nature Communications and will make an impact in the wider scientific community. A few questions and comments need to be clarified however:

Major points:

1) The authors cleverly engineered RNA mutations to determine the structural requirements for high affinity binding by the HMG domain of Sox2. They determined that Sox2-HMG interacts with A-form double stranded RNA with other RNA structural features (e.g. internal or terminal loops) only minimally affecting the binding affinity. To fully characterize the structural requirements for high affinity binding, it would be useful to determine the minimal length of dsRNA that is necessary for high affinity binding (i.e. a "minimal" binding site). In particular, what is the shortest dsRNA hairpin that is bound with high affinity? What happens if the tetraloop is removed from this shortest hairpin to make it into a fully paired RNA duplex?

2) 2D NMR spectroscopic studies are used to characterize the binding of Sox2 to DNA and RNA. The authors observed that dsDNA binding is associated to improvement of the spectral quality: because the HMG domain becomes more structured upon DNA binding, the HSQC spectrum becomes well-dispersed with uniformly narrow cross-peaks. A similar improvement is not observed upon RNA-binding, when the HSQC spectrum still contains a number of broad and poorly dispersed cross-peaks, with only a limited number of cross-peaks becoming sharper. Based on this observation, the authors suggest that the HMG domain becomes only partially ordered upon RNA binding. Line broadening observed in the RNA-bound state could be due not only to Sox2 being partially disordered, but also to the unbinding and rebinding the RNA, possibly in a different register. The authors could test whether the RNA-bound state involves multiple registers by using an RNA that corresponds to the minimal binding site (i.e. the shortest length of dsRNA that can be bound with high affinity), which would prevent rebinding in a different register.

3) What RNA and DNA sequences were used in the NMR studies? Did the authors collected HSQC spectra of Sox2-HMG bound to different RNA sequences? Did they observe spectra with similar features (i.e. with a mixture of broad and narrow lines) in each case?

4) Why didn't the authors use the chemical shift perturbations observed upon RNA-binding to characterize which residues of Sox2 interact with RNA? These data would have nicely complemented the results of their mutagenesis studies, where 48 of the 88 residues of the Sox2 HMG domain are mutated to alanine to determine their role in RNA binding.

5) From the mutagenesis studies and the observed weak effect on RNA binding affinity of mutating

H68 and H72 to Ala, the authors conclude that formation of the modest hydrophobic core in the minor wing of the HMG domain is not essential for binding. This is a strong statement based on mutagenesis of just two residues, given that we do not know the structure of Sox2-HMG bound to RNA. Based on the DNA-bound (1GT0) and apo (2LE4) structures of Sox2-HMG there are other hydrophobic residues that could be involved in the formation of a hydrophobic core in the minor wing of the domain that have not been mutated to Ala in this study, for example L64, L67, M69, V8 and P11. Mutation of H68 and H72 to Ala alone might not adequately disrupt the formation of a hydrophobic core, as these other residues may still form a hydrophobic core. Therefore, the formation of a hydrophobic core could still be important for RNA-binding.

Minor points:

1) In their study of salt dependence of Sox2 nucleic acid binding affinity, the authors observed that the electrostatic component of the free energy of binding is similar for dsDNA and RNA. However, the non-electrostatic component of the free energy of binding for RNA is more similar to that observed for ssDNA and non-specific dsDNA than that observed for dsDNA. Since the non-electrostatic component of binding has been associated to binding specificity, does this observation support the claim that RNA binding is non-specific? The authors should comment on this issue.

2) It would be useful to have a figure in the supplementary material with the structure of Sox2-HMG that highlights the major and minor wings and the residues that make important nucleotide-specific interactions.

3) The authors should state at which pH and temperature the NMR data was collected, and which specific DNA and RNA sequences were used to form the complex with Sox2-HMG.

Reviewer #3 (Remarks to the Author):

The manuscript from Holmes et al reports that the HMG domain of Sox2 can bind dsRNA with affinities 10- to 20-fold lower than that of DNA containing a sequence to which Sox2 binds in the FGF4 gene. The authors use a variety of robust biochemical assays to argue that regions within the lncRNA ES2 bind to the Sox2 HMG domain in a sequence independent manner. Based on their studies, the authors suggest two possible mechanisms by which dsRNA could alter the transcriptional activity of SOX2 in mammalian cells.

Major concerns:

The biggest deficit of this manuscript is the lack of evidence showing that the binding of dsRNA to Sox2 alters its transcriptional activity in mammalian cells. Previous studies demonstrated that Sox2 can be cross-linked to several lncRNAs in cells. However, the functional significance of this association was not determined. The authors of this study present two possible mechanisms (figure 7) to explain how dsRNA could alter the transcriptional activity of Sox2, but offer no evidence to support either model. Based on their finding that dsRNA binds in a non-sequence specific manner, the authors appear to be arguing that Sox2 transcriptional activity would change when the levels of RNA in cells drop 3- to 5-fold. Unless the authors demonstrate that changes in levels of dsRNA (or ES2 in particular) alter Sox2 function and/or binding to gene regulatory regions in specific genes, the significance of their findings is very limited.

The authors also suggest (model 2) that dsRNA could account for differences in binding of Sox2 to sites in the CCND1 promoter. However, the study they refer to argues that Sox2 binds to low affinity sites when Sox2 levels are higher in the less proliferative cells. Thus, for CCND1, previous work concluded that it is an increase in Sox2 that influences Sox2 binding to low affinity sites and

repression of the CCND1 gene. Also, since expression of CCND1 is decreasing when cells become growth arrested, RNA would need to rise to fit the second possible mechanism offered in figure 7, which is the opposite of their argument that RNA goes down in growth arrested cells.

Another conceptual issue is that this study focuses on Sox2 in the absence of other transcription factors, such as Oct4 (see below), which bind to adjacent DNA sites along with Sox2 in the FGF4 enhancer and many other genes. Could adding Oct4 alter the outcome of some of their assays and the interpretation of their findings? In this case, they would need to use a DNA sequence containing both the Sox2 and Oct4 binding sequence. Finally, it is unclear whether the authors are largely discounting a role for sequence specific actions of lncRNA, such as ES2, over the transcriptional activity of Sox2.

Technical concerns:

1. The authors state that they are studying a Sox2 binding site present in the FGF4 promoter. The site in the FGF4 gene that they are referring to is in the FGF4 enhancer located within an exon of the FGF4 gene. This sequence works in conjunction with the adjacent Oct4 binding sequence to provide strong expression of FGF4. It would have also been helpful for the authors to provide the specific locations of Sox2 binding sites within the five genes listed in Fig S1.

2. The sequence used in their studies contains a Sox2 site and non-specific flanking sequences. Would they have observed similar results in their competition assay if the dsDNA used contained the Sox2:Oct4 sequence and the assay performed in the presence of Sox2 and Oct4? Sox2 in many, if not most cases, binds to DNA in conjunction with other transcription factors.

3. Are the dsRNA regions and loops shown in figure 2 predicted or verified?

4. The second slower migrating complex observed in their EMSA binding assays (fig 4b) is described as a non-specific binding event (page 10 line 200-201). How do the authors envision this occurring? Sox2 has been reported to be capable of dimerizing. Also, did the authors include poly dG/dC in the EMSA mix, since this reduces non-specific binding of SOX2 to DNA fragments? Would adding poly dG/dC alter the binding affinity of SOX2 to dsRNA and dsDNA equally?

5. The detailed biochemical studies were tested with only a single lncRNA. Performing at least some of their biochemical studies with a second lncRNA would have made their findings even more convincing.

6. The authors claim that the Sox2 sequence (mouse or human?) is provided in Supplemental Table S8. This table does not provide that information.

Dear Editor and Reviewers:

We would like to thank you for your time and effort to review and assess the quality of our manuscript. We appreciate the support of the three reviewers for publication of this work after some concerns are addressed. In response, we have made major changes to the manuscript, including execution of a series of *in vivo* experiments and associated analysis. In addition to this experimental work, we have made numerous changes to the manuscript to improve the narrative, as suggested by the reviewers. A detailed point-by-point response to the reviews is below, with our response in bold italics. We believe with this extensive revision that we have addressed all of the concerns raised in review in full.

Reviewer #1 (Remarks to the Author):

The authors make a compelling biochemical case for the ability of the Sox2 protein to bind double stranded RNA, in addition to its normal double stranded DNA target. This is an important observation, since Sox2 is normally thought of as a transcription factor, such that effect it exerts in cells would be attributed to that function. Certainly I am convinced that Sox2 can associate with double-stranded RNA, but I'm left with many questions regarding the optimal RNA target.

Additional details about HMG-box proteins would put these results in perspective. There appears to be a consensus that HMG-box proteins prefer to bind to non-canonical B-form DNAs. In particular, they bind to kinked or unwound duplex DNA (noting, however, that the crystal structure 1GT0 used in Figure 6 contains a normal dsDNA, although Sox2 is bound at the blunt end of the duplex which looks as if it is unwound). Also, they are known to introduce a bend in duplex DNA. With these details in mind, the ability of Sox2 to bind dsRNA is still surprising, but of all DNA binding proteins, HMG-boxes might be the most likely to bind.

We have addressed these points in a modified discussion.

The authors assume that the reader is familiar with the architecture of HMG boxes. For example, they refer to the major and minor wings of the protein, but they do not show a protein structure and identify these elements. The structures shown in Figure 6 from the cocrystals structure do not help the reader, since this surface rendering obscures the juxtaposition of the α-helices and the position of the tails. Please provide another figure that explicitly shows the protein's elements.

We appreciate the request for more context to follow the structural analysis, we now provide a figure in which the major and minor wings of the Sox2-HMG domain in complex with DNA have been annotated (Supplementary Fig. S6) as well as labeling the major and minor wings of the Sox2-HMG domain in Fig. 6.

Questions about RNA targets and relative binding affinities:
1. Sox2 seems to prefer an RNA duplex with an internal bulge, rather than a perfect duplex (Figure 3c).

We disagree with this statement. The differences between the measured K_D values in Fig. 3c are very small, ranging from 10 to 15 nM, which we consider nearly identical ($\Delta\Delta G = 1.0$ kJ mol^{-1}). In general, we choose to be conservative in our analysis of differences in

binding affinity and do not generally interpret differences in K_D less than 4-fold, which correspond to a $\Delta\Delta G$ = 3.4 kJ mol^{-1}. That being said, we agree that our conclusion that Sox2 prefers double-stranded RNA is too strong in that it clearly binds small hairpins that are either perfect duplexes or have internal loops with nearly identical affinities. We have thus moderated this claim throughout the paper, including a revised title, to state that "the Sox2-HMG prefers RNA elements that include double-stranded features."

The nucleic acid targets for Sox2 in Figure 3 need more descriptions: why, for example, does Sox2 in 3c,d bind to the hairpin with a perfect duplex (17 base pairs) with KD=15 nM, but in 3f to an RNA duplex with KD,app = 35 nM? (The authors explain results in 3d as due to poor annealing of the two RNA strands).

Again, we submit that these differences are quite small—just over 2-fold which is from an energetic perspective quite small. We acknowledge that the explanation given (poor annealing of strands) is not likely to be correct. Instead, since the exact preferences for Sox2-HMG binding to RNA are not known, it is more likely that this difference is due to small differences in the intermolecular interactions between the two RNAs.

The authors suggest (ll 152-155) that other structural features could contribute to Sox2 binding, which is supported by their data, but also agrees with what is known about the imperfect dsDNAs that Sox2 prefers. More emphasis on this preference and how it translates to the typically short and imperfect RNA duplexes in RNA structures could be made.

We agree, and as stated above, our conclusions now reflect a broader definition of the preferred Sox2 binding sites that include non-canonical helical features.

2. What RNAs were used in S4 and S5? Which nucleic acids were added to Sox2 HMG in the NMR experiments?

We thank the reviewer for pointing out that omission. We used the FGF4 dsDNA, Loop B RNA Bulge(0+1) and Loop B RNA, fully paired (Fig. 3c) for our NMR experiments. This information for the DNA and RNA substrates has been added to the text and to the legend of Fig. 5.

3. Do the authors have any ideas about the RNA binding site size for Sox2? I imagine that in the RNA hairpins with an internal bulge, Sox2 is sitting on the duplex between hairpin loop and bulge, to maximize its contact with a deformed A-form duplex.

We do not know what the minimal RNA binding site for Sox2-HMG is from the experimental data presented in the original manuscript? paper. We agree with this reviewer that the Sox2-HMG domain restricts its contacts to mostly double stranded regions. To address this question directly, we have performed additional binding experiments with a set of hairpins derived from Loop B RNA (Full Repair) that comprise 5, 9 and 13 base pairs. While we saw a ~3-fold modest reduction in binding affinity for these hairpins (data presented in Figure 3d), the smallest of the hairpins still binds Sox2-HMG domain with reasonable affinity (72 nM). We have included a description of these results in the section "Sox2 is a double-stranded RNA-binding protein."

4. A concern in Figure 2c: they monitor the 3'fluorophore during binding, and fit to a 1:1 binding isotherm. The concentration of RNA (3 nM) is very close to their measured KD; did they repeat these experiments at lower RNA concentrations? Did they measure stoichiometry?

Yes, this is a concern. Because of instrument sensitivity and the ~20% efficiency in 3'-end labeling of RNA using the periodate oxidation method, we could not routinely use concentrations lower than 3 nM of RNA. To address this issue, we did use a two-state transition binding equation that accounts for ligand depletion (described in methods section "Binding measured by fluorescence anisotropy.". These K_Ds agree with similar measurements by EMSA that can use significantly lower ^{32}P-labeled RNA concentrations, but suffer other issues (i.e., it is a non-equilibrium binding measurement) that led us to use FA for all quantitative measurements. For example, the "Fully paired" RNA (Fig. 3c) gives a K_D of 15 ± 2 nM by FA and 20 ± 4 nM by EMSA. Similarly, other select RNAs yielded K_D values that are within 2-fold in each method.

Further, we did measure the stoichiometry of the interaction (example provided in Fig. 3b) and observed two distinct transitions with correspond to 1:1 RNA:protein and 1:2 RNA:protein. This corresponds to the two transitions that we observe by fluorescence anisotropy. All $K_{D,\,app}$s reported correspond to the first transition.

5. A peculiarity: Competition assays are described (ll 474-475) as equilibrating overnight. Is overnight at room temp necessary? If so, how do the authors explain this? Conformational changes of nucleic acids or Sox2? Incredibly slow off-rates (not consistent with the KD)?

Preliminary competition experiments equilibrated for 1 hour at room temperature did not reach equilibrium as neither DNA-RNA competition nor RNA-RNA self-competition experiments had measurable IC_{50}s. Although the reactions reached equilibrium overnight at room temperature, this likely exceeds the minimum equilibration time and instead guaranteed that equilibration had been reached. This behavior has been seen before in similar types of experiments. For example, in a study by Pfingsten et al. ("Mutually exclusive binding of telomerase RNA and DNA by Ku alters telomerase recruitment model" Cell 2012), the authors had to perform similar overnight incubations to ensure that the reaction had come to full equilibrium. Since we do not know any of the association or dissociation rates of Sox2-HMG for DNA or RNA, we cannot comment as to the specific reason why long incubation times were required.

6. Figure 6a with the DNA duplex bound is from the cocrystals. Have the authors tried to model in an A-form duplex? Not easy to do, but it could be informative.

No, we have not done so. It is clear from the NMR data that the HMG domain bound to the RNA is different, but we don't have any idea what those structural differences are and so we do not have a good starting point for modeling the protein-RNA complex. As structural biologists, getting an experimentally based structure of the complex is of high priority and will certainly answer many questions posed by this study. However, determination such a structure is beyond the scope of this study.

7. Figure 5 is quite striking – is there any reason to expect that the tails of the protein are not

involved with RNA binding?

We agree, which is why the data is included. As noted in the discussion section, it is well known that the HMG domain of Sox2 and related proteins (such as LEF1) are loosely structured in their apo- state. Further, detailed studies of LEF1 (reference 49) suggest that the major wing is more organized than the minor wing that consists of the tails. It is certainly tempting to speculate that the tails may not be involved in RNA binding, but we do see contributions from both wings (including the tails) in the alanine scan (Fig. 6b, grey versus white shading). The participation of this structural element in RNA recognition will be addressed in future structural analysis.

Reviewer #2 (Remarks to the Author):

This manuscript reports the results of a study on the transcription factor Sox2. The main focus of the work is the characterization of the interaction of Sox2 with RNA. The authors observe that Sox2 directly binds to RNA using its high mobility group (HMG) DNA-binding domain. In addition, they report that RNA recognition occurs through binding to double stranded RNA (dsRNA) in a non-specific fashion. These results indicate that interaction of Sox2 with RNA may play an important role in how Sox2 regulates gene expression.

This manuscript is an interesting and rigorous study that provides substantial new information to understand a novel and, so far, not well characterized interaction of Sox2 with RNA that is potentially very important for its function. For these reasons I believe it will be interesting to the readers of Nature Communications and will make an impact in the wider scientific community. A few questions and comments need to be clarified however:

Major points:

1) The authors cleverly engineered RNA mutations to determine the structural requirements for high affinity binding by the HMG domain of Sox2. They determined that Sox2-HMG interacts with A-form double stranded RNA with other RNA structural features (e.g. internal or terminal loops) only minimally affecting the binding affinity. To fully characterize the structural requirements for high affinity binding, it would be useful to determine the minimal length of dsRNA that is necessary for high affinity binding (i.e. a "minimal" binding site). In particular, what is the shortest dsRNA hairpin that is bound with high affinity? What happens if the tetraloop is removed from this shortest hairpin to make it into a fully paired RNA duplex?

We agree this is a key concern (raised by 2 reviewers) and have addressed it with new experimental data. Please see our response to Reviewer 1, point 3, which addresses this comment.

2) 2D NMR spectroscopic studies are used to characterize the binding of Sox2 to DNA and RNA. The authors observed that dsDNA binding is associated to improvement of the spectral quality: because the HMG domain becomes more structured upon DNA binding, the HSQC spectrum becomes well-dispersed with uniformly narrow cross-peaks. A similar improvement is not observed upon RNA-binding, when the HSQC spectrum still contains a number of broad and poorly dispersed cross-peaks, with only a limited number of cross-peaks becoming sharper.

Based on this observation, the authors suggest that the HMG domain becomes only partially ordered upon RNA binding. Line broadening observed in the RNA-bound state could be due not only to Sox2 being partially disordered, but also to the unbinding and rebinding the RNA, possibly in a different register. The authors could test whether the RNA-bound state involves multiple registers by using an RNA that corresponds to the minimal binding site (i.e. the shortest length of dsRNA that can be bound with high affinity), which would prevent rebinding in a different register.

We agree with the reviewer that it is highly likely that the HMG domain is binding in multiple registers to the RNA substrates and testing a minimal substrate to address this issue is an excellent suggestion. As part of revising the manuscript, we have found that a minimal RNA hairpin of 5 base pairs binds to the Sox2-HMG domain with moderately high affinity (Fig. 3d). We would, however, like to reserve further structural analysis of the Sox2-HMG domain with RNA for a future study that will more thoroughly and rigorously address this point that we believe is beyond the scope of this initial report.

3) What RNA and DNA sequences were used in the NMR studies? Did the authors collected HSQC spectra of Sox2-HMG bound to different RNA sequences? Did they observe spectra with similar features (i.e. with a mixture of broad and narrow lines) in each case?

With respect to the point regarding the identity of the ligands used in our structural studies, we apologize for and have corrected the omission, please refer to the response to reviewer 1, comment 2. Regarding the collection of HSQC data on different RNAs, as pointed out in the text, we did investigate two that are related in sequence with the exception that one contains a bulged adenosine (Loop B, Bulge(0+1)) and is a fully base paired hairpin. Both spectra showed nearly identical features with respect to a mixture of broad and narrow lines. While line broadening can be due to a number of kinetic features, including rebinding in alternate modes, we don't favor that interpretation due to the similarity in K_D***s for RNA and DNA, which suggest a similar off rate. Also, the serious line-broadening of the free state is consistent with an unfolded state, partial ordering on binding is consistent with our observations.***

4) Why didn't the authors use the chemical shift perturbations observed upon RNA-binding to characterize which residues of Sox2 interact with RNA? These data would have nicely complemented the results of their mutagenesis studies, where 48 of the 88 residues of the Sox2 HMG domain are mutated to alanine to determine their role in RNA binding.

Although there are assignments for Sox2-HMG bound to a DNA target in complex with the POU domain of Oct4, unfortunately we were unable to transfer these assignments to our Sox2-HMG-DNA spectra, presumably due to the impact of the bound POU domain on the chemical shifts of Sox2-HMG. Additionally, the chemical shifts of the free Sox2-HMG and Sox2-HMG-RNA complex are unassigned and the assignment of these resonances is beyond the scope of this study. We plan on complementing this study with a structural study in the future.

5) From the mutagenesis studies and the observed weak effect on RNA binding affinity of mutating H68 and H72 to Ala, the authors conclude that formation of the modest hydrophobic core in the minor wing of the HMG domain is not essential for binding. This is a strong statement

based on mutagenesis of just two residues, given that we do not know the structure of Sox2-HMG bound to RNA. Based on the DNA-bound (1GT0) and apo (2LE4) structures of Sox2-HMG there are other hydrophobic residues that could be involved in the formation of a hydrophobic core in the minor wing of the domain that have not been mutated to Ala in this study, for example L64, L67, M69, V8 and P11. Mutation of H68 and H72 to Ala alone might not adequately disrupt the formation of a hydrophobic core, as these other residues may still form a hydrophobic core. Therefore, the formation of a hydrophobic core could still be important for RNA-binding.

This point is well taken and we have modified the language of the text to specifically state that we only tested these two residues in the minor wing. We make no specific statement about the folding of the minor wing based upon these two mutations, but our data remains clear with respect to the folding of the major wing.

Minor points:

1) In their study of salt dependence of Sox2 nucleic acid binding affinity, the authors observed that the electrostatic component of the free energy of binding is similar for dsDNA and RNA. However, the non-electrostatic component of the free energy of binding for RNA is more similar to that observed for ssDNA and non-specific dsDNA than that observed for dsDNA. Since the non-electrostatic component of binding has been associated to binding specificity, does this observation support the claim that RNA binding is non-specific? The authors should comment on this issue.

We agree with this insightful point, indeed the non-specific component supports the notion that Sox2-HMG recognizes a broad range of RNA ligands. This is also consistent with our NMR data that suggests HMG remains in partially ordered/disordered state upon binding RNA, especially since same observations are made for both the Bulge(0+1) and Fully Repaired RNAs. A sentence at the end of the section describing the electrostatics of binding has been added that directly addresses this point.

2) It would be useful to have a figure in the supplementary material with the structure of Sox2-HMG that highlights the major and minor wings and the residues that make important nucleotide-specific interactions.

We agree and now provide the requested figure in which the major and minor wings of the Sox2-HMG domain in complex with DNA have been annotated (Supplementary Fig. S6).

3) The authors should state at which pH and temperature the NMR data was collected, and which specific DNA and RNA sequences were used to form the complex with Sox2-HMG.

Our apologies about that omission. We used the FGF4 dsDNA and Loop B RNA Bulge(0+1) (Fig. 3c) for our NMR experiments. This information for the DNA and RNA substrates has been added to the text and to the legend of Fig. 5. The spectra were collected at pH 6.5 and 25 °C; this information has been added to the methods.

Reviewer #3 (Remarks to the Author):

The manuscript from Holmes et al reports that the HMG domain of Sox2 can bind dsRNA with affinities 10- to 20-fold lower than that of DNA containing a sequence to which Sox2 binds in the FGF4 gene. The authors use a variety of robust biochemical assays to argue that regions within the lncRNA ES2 bind to the Sox2 HMG domain in a sequence independent manner. Based on their studies, the authors suggest two possible mechanisms by which dsRNA could alter the transcriptional activity of SOX2 in mammalian cells.

Major concerns:

The biggest deficit of this manuscript is the lack of evidence showing that the binding of dsRNA to Sox2 alters its transcriptional activity in mammalian cells. Previous studies demonstrated that Sox2 can be cross-linked to several lncRNAs in cells. However, the functional significance of this association was not determined. The authors of this study present two possible mechanisms (figure 7) to explain how dsRNA could alter the transcriptional activity of Sox2, but offer no evidence to support either model. Based on their finding that dsRNA binds in a non-sequence specific manner, the authors appear to be arguing that Sox2 transcriptional activity would change when the levels of RNA in cells drop 3- to 5-fold. Unless the authors demonstrate that changes in levels of dsRNA (or ES2 in particular) alter Sox2 function and/or binding to gene regulatory regions in specific genes, the significance of their findings is very limited.

The authors also suggest (model 2) that dsRNA could account for differences in binding of Sox2 to sites in the CCND1 promoter. However, the study they refer to argues that Sox2 binds to low affinity sites when Sox2 levels are higher in the less proliferative cells. Thus, for CCND1, previous work concluded that it is an increase in Sox2 that influences Sox2 binding to low affinity sites and repression of the CCND1 gene. Also, since expression of CCND1 is decreasing when cells become growth arrested, RNA would need to rise to fit the second possible mechanism offered in figure 7, which is the opposite of their argument that RNA goes down in growth arrested cells.

Another conceptual issue is that this study focuses on Sox2 in the absence of other transcription factors, such as Oct4 (see below), which bind to adjacent DNA sites along with Sox2 in the FGF4 enhancer and many other genes. Could adding Oct4 alter the outcome of some of their assays and the interpretation of their findings? In this case, they would need to use a DNA sequence containing both the Sox2 and Oct4 binding sequence. Finally, it is unclear whether the authors are largely discounting a role for sequence specific actions of lncRNA, such as ES2, over the transcriptional activity of Sox2.

The concerns raised by this reviewer primarily arise from the discussion in which we attempt to provide mechanistic explanations for our observation of RNA binding by Sox2 in vitro. We realize that this is certainly an overreach and we have no data to support these discussion points.

We have taken the main issue raised by this reviewer very seriously and have conducted a new line of investigation to address it. With respect to the concern about functional experiments, we believe that what this reviewer is asking for is whether Sox2 interacts with RNA in vivo in support of the in vitro binding analysis. To address this, we have initiated a collaboration with Prof. John Rinn and conducted two types of RIP

experiments using a highly specific and well validated Sox2 antibody (Supplementary Figure S10). We performed four independent replicates of a formaldehyde crosslinked RIP experiment and four independent replicates of a UV-RIP experiment in mouse embryonic stem cells (mESCs). The UV crosslinking strategy is generally considered to be highly selective for protein-RNA crosslinks that reflect direct protein-nucleic acid interactions. Notably, we found a substantial number of high-confidence genes and that the ~75% of the UV-crosslinked RNAs were also found in the fRIP analysis. These data clearly indicate that Sox2 is associated with a substantial number of transcripts in vivo, in support of the principal claim of this study that Sox2 is an RNA binding protein. The results of these experiments have been added to the revised manuscript (Figure 7).

We should further note to this reviewer that we are refraining from making further claims about the nature of specific Sox2-RNA interactions in the discussion at this time in order to keep the focus of the study on RNA binding. Certainly, now that we have firmly established the presence of Sox2-RNA interactions in a biologically relevant context, future studies will center upon understanding how RNA binding affects Sox2 function.

Technical concerns:

1. The authors state that they are studying a Sox2 binding site present in the FGF4 promoter. The site in the FGF4 gene that they are referring to is in the FGF4 enhancer located within an exon of the FGF4 gene. This sequence works in conjunction with the adjacent Oct4 binding sequence to provide strong expression of FGF4. It would have also been helpful for the authors to provide the specific locations of Sox2 binding sites within the five genes listed in Fig S1.

We thank the reviewer for pointing that out, we now refer to the sequence as the enhancer. The Sox2 binding sites are now shown in bold in S1.

2. The sequence used in their studies contains a Sox2 site and non-specific flanking sequences. Would they have observed similar results in their competition assay if the dsDNA used contained the Sox2:Oct4 sequence and the assay performed in the presence of Sox2 and Oct4? Sox2 in many, if not most cases, binds to DNA in conjunction with other transcription factors.

The reviewer is correct in that Sox2 associates with Oct4 and other factors when it is bound to it promoter sequence. However, as a pioneer transcription factor, it is likely to also be found in isolation on DNA promoters prior to binding of other transcription factors including Oct4. We would be very surprised if the results of competition assays with both Sox2 and Oct4 assembled on a promoter were not different. However, since the focus of this paper is on RNA binding, we believe that these experiments are better suited for a study that focuses on the mechanistic impacts of the ability of Sox2 to bind RNA.

3. Are the dsRNA regions and loops shown in figure 2 predicted or verified?

These loops are predicted by Mfold. As nucleic acid structural biologists, we fully realize that the bases within internal loops will stack into the helix and form non-canonical base pairs, but the exact pairing scheme cannot be computationally predicted, so we leave them denoted as an "unpaired internal loop." This does not change our conclusions

since Sox2-HMG binds very different internal loops (e.g., purine-rich and pyrimidine-rich) or small bulges with the same affinity, indicating that the nature of the loop is not important for binding.

4. The second slower migrating complex observed in their EMSA binding assays (fig 4b) is described as a non-specific binding event (page 10 line 200-201). How do the authors envision this occurring? Sox2 has been reported to be capable of dimerizing. Also, did the authors include poly dG/dC in the EMSA mix, since this reduces non-specific binding of SOX2 to DNA fragments? Would adding poly dG/dC alter the binding affinity of SOX2 to dsRNA and dsDNA equally?

The issue of the slower migrating, secondary complex in the EMSA binding assay has been addressed in a few ways.

We favor assignment of the 2nd band to a 2nd, non-specific binding event for two reasons. The first is based on literature precedent, a smFRET study of Sox2's binding (Moosa, 2018; IJMS) clearly reveals Sox2 binds to a non-canonical site at high concentrations. Second, when investigating binding to the minimized ligand such that it can only accommodate a single Sox2-HMG we no longer observe the second binding event. This strongly suggests that it is nucleic acid dependent and not a protein/protein dimerization event.

As the reviewer suggests, we did explore the addition of a variety of "non-specific" DNAs including salmon testes DNA, poly dGdC, and tRNA in our reaction conditions to measure K_D, but they all reduced Sox2-HMG's affinity for specific DNA. Thus, we decided to not use these "competitor" nucleic acids in any of our experiments.

5. The detailed biochemical studies were tested with only a single lncRNA. Performing at least some of their biochemical studies with a second lncRNA would have made their findings even more convincing.

We argue that a sufficiently diverse set of model RNAs (Supplementary Figure 3) beyond ES2 was tested. That fact that in all cases where the RNA had some double-stranded character we observed similar binding affinities strongly supports our conclusion that binding is non-sequence specific, but rather structure selective. Testing binding in the other implicated lncRNA, RMST, was precluded by the intractable size of that lncRNA.

6. The authors claim that the Sox2 sequence (mouse or human?) is provided in Supplemental Table S8. This table does not provide that information.

We thank the reviewer for noting this omission. The human Sox2 sequence is in Supplemental Table S9.

REVIEWERS' COMMENTS:

Reviewer #1 (Remarks to the Author):

This revised manuscript is a more than adequate revision, and is now an exciting advance in the field. The demonstration of Sox2 binding to RNA in vitro and in cellulo firmly demonstrates a new paradigm for transcription factor regulation. The study is comprehensive, everything one would want in characterizing RNA:protein interactions, and has found a particularly intriguing result by NMR that certainly deserves more attention (beyond the scope of this manuscript).

Paper is well written, easy to read, and the Figures are informative and clear.

Kathleen B Hall, Ph.D.

Reviewer #3 (Remarks to the Author):

The revised manuscript from Holmes et al has addressed adequately the main concerns raised by this reviewer. Of particular note is the inclusion of new data (figure 7) showing that Sox2 can be crossed-linked to RNA in mESC. This is an important addition to their study. Although the authors do not show that this association of Sox2 with RNA in vivo alters the function of Sox2, this reviewer agrees that this issue could be the subject of a future paper. In regard to the technical issues raised, the responses of the authors to this reviewer are adequate. However, it is surprising that adding poly dG/dC reduced Sox2-HMG’s specific binding to DNA.
